# Expansion of sweet taste receptor genes in grass carp (*Ctenopharyngodon idellus*) coincided with vegetarian adaptation

**DOI:** 10.1186/s12862-020-1590-1

**Published:** 2020-02-11

**Authors:** Xiao-Chen Yuan, Xu-Fang Liang, Wen-Jing Cai, Shan He, Wen-Jie Guo, Kang-Sen Mai

**Affiliations:** 1 College of Fisheries, Chinese Perch Research Center, Huazhong Agricultural University, No.1, Shizishan Street, Hongshan District, Wuhan, 430070 Hubei Province China; 2 Innovation Base for Chinese Perch Breeding, Key Lab of Freshwater Animal Breeding, Ministry of Agriculture, Wuhan, 430070 China; 3 The Key Laboratory of Aquaculture Nutrition and Feeds, Ministry of Agriculture, The Key Laboratory of Mariculture, Ministry of Education, Ocean University of China, Qingdao, 266003 Shandong China

**Keywords:** Taste receptor, Gene expansion, Adaptive evolution, Food habit transition, Herbivory

## Abstract

**Background:**

Taste is fundamental to diet selection in vertebrates. Genetic basis of sweet taste receptor in the shaping of food habits has been extensively studied in mammals and birds, but scarcely studied in fishes. Grass carp is an excellent model for studying vegetarian adaptation, as it exhibits food habit transition from carnivory to herbivory.

**Results:**

We identified six sweet taste receptors (*gcT1R2A-F*) in grass carp. The four *gcT1R2s* (*gcT1R2C-F*) have been suggested to be evolved from and paralogous to the two original *gcT1R2s* (*gcT1R2A* and *gcT1R2B*). All gcT1R2s were expressed in taste organs and mediated glucose-, fructose- or arginine-induced intracellular calcium signaling, revealing they were functional. In addition, grass carp was performed to prefer fructose to glucose under a behavioral experiment. Parallelly, compared with gcT1R2A-F/gcT1R3 co-transfected cells, gcT1R2C-F/gcT1R3 co-transfected cells showed a higher response to plant-specific fructose. Moreover, food habit transition from carnivory to herbivory in grass carp was accompanied by increased gene expression of certain *gcT1R2s*.

**Conclusions:**

We suggested that the gene expansion of *T1R2s* in grass carp was an adaptive strategy to accommodate the change in food environment. Moreover, the selected gene expression of *gcT1R2s* might drive the food habit transition from carnivory to herbivory in grass carp. This study provided some evolutional and physiological clues for the formation of herbivory in grass carp.

## Background

Taste perception, conveying important dietary information, is fundamental for the survival of animals ranging from insects to mammals [1, 2]. All tastes are combinations of five basic modalities: sweet, umami, bitter, salty, and sour [3, 4]. Sensory systems display remarkable flexibility across vertebrates, with some abandoning unnecessary sensory modalities [5–7] while others evolving new adaptive sensory modalities [8]. In vertebrates, sweet and umami tastes are identified by a class of G protein-coupled receptors (GPCRs) termed taste receptor type 1 (T1R) [2]. Most vertebrates have three T1Rs, with the T1R1-T1R3 heterodimer mediating umami taste and the T1R2-T1R3 heterodimer mediating sweet taste [2, 9].

Taste perception varies enormously across different lineages and species of vertebrates [1, 2]. Sweet taste “blindness” observed in some carnivorous mammals, such as domestic cat, California sea lion, southern fur seal, Pacific harbor seal, Asian small-clawed otter, spotted hyena, fossa, banded linsang, bottlenose dolphin, and vampire bats, have been suggested to be the consequence of pseudogenization of *T1R2* since they do not require the receptor for sweet food perception [6, 7, 10, 11]. However, almost all omnivorous and herbivorous mammals with the habit to consume sugars have a functional *T1R2* structure [6, 7, 10, 12–15]. Unlike pseudogenization of *T1R2* in some carnivorous mammals, the evolution of sweet taste in birds is in absence of *T1R2* despite food habits [16–18]. Nevertheless, ancestral umami taste receptor has been repurposed to detect sweet in the hummingbird [17]. The absence or presence of intact *T1R2* is concordant with food habits in mammals and birds, suggesting the adaption of *T1R2* evolution to food habit formation and environmental change.

Teleost fishes represent about half of all living vertebrate species and provide important models for evolutionary study [19]. The food habits of fish, which are more sensitive to water-soluble chemicals than mammals, are demonstrated to be associated with chemosensory-mediated taste sense [20, 21]. Unlike pseudogenization or absence in mammals or birds, most fish have been shown to possess two or three *T1R*2s [16, 22, 23]. Whether the gene number of fish *T1R2* genes related to the formation of food habits like mammals or birds is worthy of further exploration [24].

Constituting a member of the Cyprinidae family, grass carp (*Ctenopharyngodon idellus*) is an excellent model for studying the formation mechanism of herbivory as it shows the food habit transition from carnivory to herbivory [25, 26]. Grass carp is carnivorous when its total length is shorter than 3 cm, then fish of 3–5.5 cm undergo the food transition stage from zooplankton or benthos to aquatic macrophytes, whereas fish larger than 5.5 cm is completely herbivorous [25–27]. We hypothesized *T1R2* gene might be involved in food habit transition and adaptation to a vegetarian diet observed in grass carp. For this purpose, six grass carp *T1R2s* (*gcT1R2s*) were identified from draft genome sequences and their evolutionary analysis was conducted among fish with different food habits. When transfected in Human embryonic kidney 293 T (HEK293T) cells, gcT1R2/gcT1R3 responded to ubiquitous glucose and plant-specific fructose in intracellular calcium signaling. Gene expressions of *T1R2s* in grass carp before and after food transition from carnivory to herbivory were also investigated. This study might provide new insights into the adaptive evolution of sweet taste receptors during food habit formation in fish.

## Results

### Characterization of *gcT1R2* genes

**Fig. 1:**
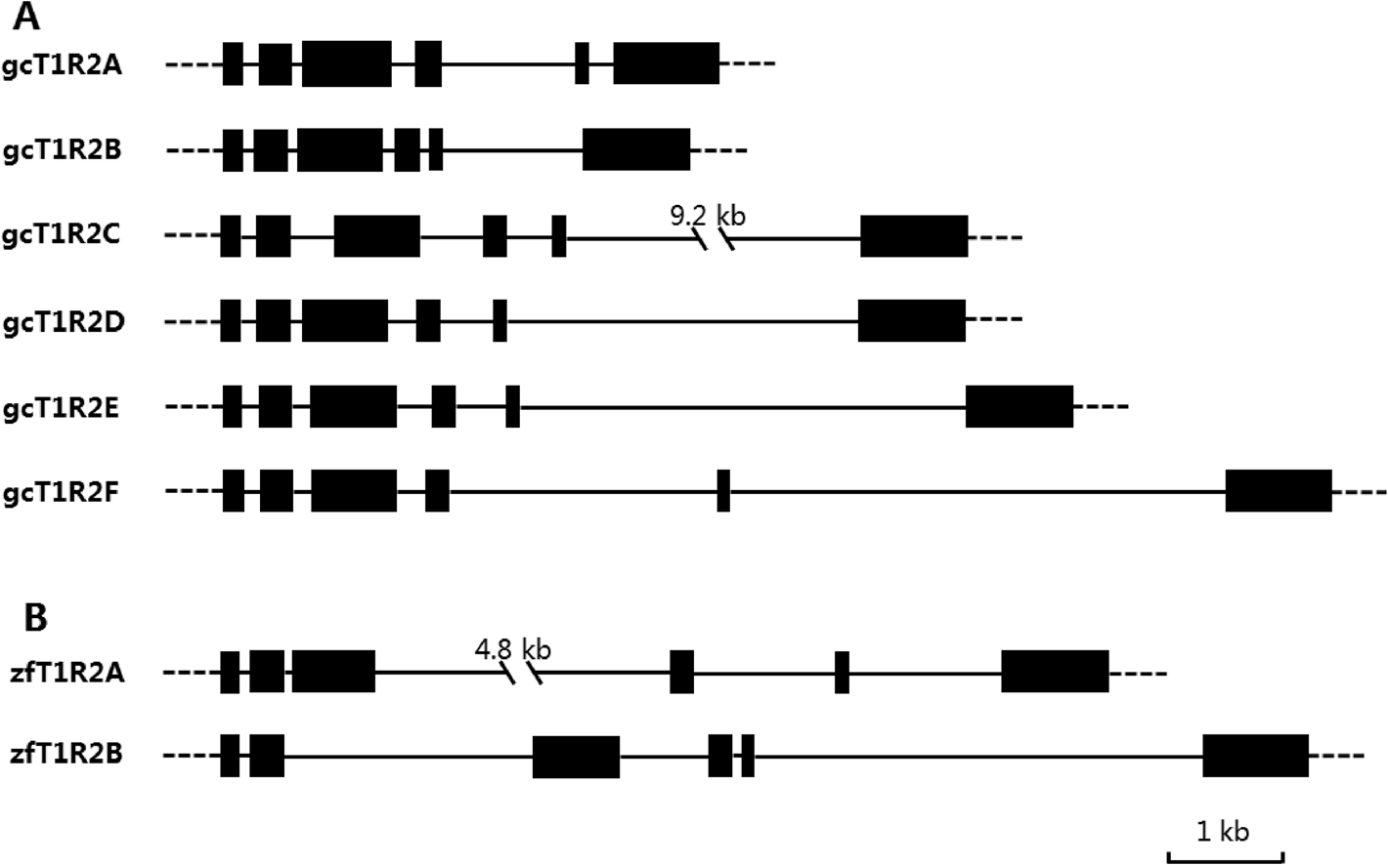
The gene structures of *T1R2* genes in grass carp (**a**) and zebrafish (**b**). The black lines indicate introns, and the black boxes indicate exons. The six *gcT1R2* genes we obtained contained 6 exons and 5 introns as well as the genomic structure of two zebrafish *T1R2* genes

Conducting a homology search, we sequenced six T1R2 genes, named gcT1R2A-F, from the grass carp genome sequence (Genbank accession no. in Table 1 and detail information in electronic Additional file 1: Dataset S1). The six T1R2s of grass carp showed higher than 78% identities with each other, higher than 73% identities with two T1R2s of zebrafish, whereas lower than 35% identities with human T1R2 (electronic Additional file 5: Table S4). The genomic structure of all *gcT1R2s* consisted of six coding exons and five introns like zebrafish *T1R2s* (Fig. 1).

**Table 1:** Numbers of sweet taste receptors and pseudogenes in fishes

Order	Species	Food habits	Numbers of T1R2	Accession no.
Coelacanthiformes	Coelacanth (*Latimeria chalumnae*)	Carnivore	2	XM_005986116 XM_005986117
Lepidosteiformes	Spotted gar (*Lepisosteus oculatus*)	Carnivore	2	XM_015337079 XM_015337086
Characiformes	Cavefish (*Astyanax mexicanus*)	Carnivore	2	ENSAMXG00000014366 ENSAMXG00000014380
Gasterosteiformes	Stickleback (*Gasterosteus aculeatus*)	Carnivore	8	ENSGACG00000006506 ENSGACG00000006510 ENSGACG00000006517 ENSGACG00000006525 ENSGACG00000006529 ENSGACG00000006534 ENSGACG00000006536 ENSGACG00000006762
Perciformes	European seabass (*Dicentrarchus labrax*)	Carnivore	2	DLAgn_00132730 DLAgn_00132740
Cyprinodontiformes	Guppy (*Poecilia reticulata*)	Omnivore	2	XM_008414738.2 XM_008413473.2
Cyprinodontiformes	Southern platyfish (*Xiphophorus maculatus*)	Omnivore	2	XM_005800149.1 XM_023341410.1
Beloniformes	Medaka (*Oryzias latipes*)	Omnivore	3	AB200906 AB200907 AB200908
Perciformes	Zebra mbuna (*Maylandia zebr*a)	Omnivore	2	XM_004554287.4 XM_004554097.2
Perciformes	Tilapia (*Oreochromis niloticus*)	Omnivore	3	XM_005478384 XM_003444871 XM_003444784
Tetraodontiformes	Fugu (*Takifugu rubripes*)	Omnivore	2	AB200911 AB200912
Cypriniformes	Common carp (*Cyprinus carpio*)	Omnivore	2	XM_019093519.1 XM_019091545.1
Cypriniformes	Zebrafish (*Danio rerio*)	Omnivore	2	NM_001039831 NM_001083856
Cypriniformes	Blunt snout bream (*Megalobrama amblycephala*)	Herbivore	3	SRP090157
Cypriniformes	Grass carp (*Ctenopharyngodon idellus*)	Herbivore	6	KU976430 KU976431 KU976432 KU976433 KU976434 KU976435

Although several carnivorous and omnivorous fish species have two or three *T1R2* genes, *T1R2* genes have been highly duplicated in grass carp (Table 1). Moreover, among Cypriniformes, highly gene duplication of T1R2s could only be observed in herbivorous grass carp and blunt snout bream.

### Evolutionary analyses of gcT1R2s

**Fig. 2:**
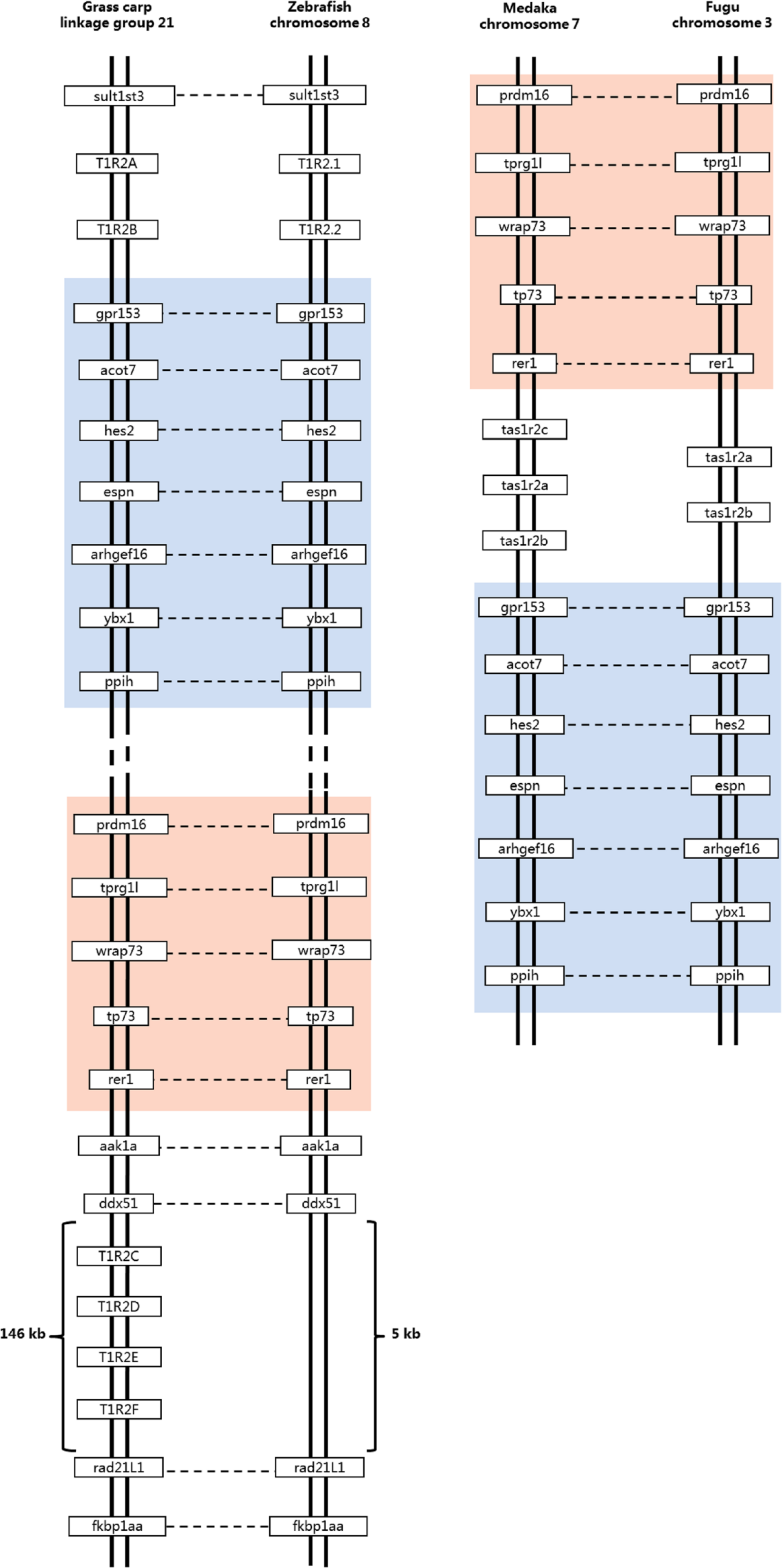
Synteny analysis of *T1R2* genes. The synteny analysis performed by searching gene(s) flanking *T1R2* in genomes of zebrafish, medaka and fugu using map viewer of NCBI. GenBank accession numbers of the adjacent genes of zebrafish *T1R2.1* (NM_001039831.1) and *T1R2.2* (NM_001083856.1) in the figure were as follows: *sult1st3*, NM_183348.2; *gpr153*, XM_009304261.1; *acot7*, NM001004617.1; *hes2*, NM_001045353.1; *espn*, NM_001123282.1; *arhgef16*, NM_001123283.1; *ybx1*, NM_001126457.1; *ppih*, NM_001009902.2; *prdm16*; XM_005167301.2; *tprg1l*, XM_001922766.5; *wrap73*, NM_199893.1; *tp73*, NM_183340.1; *rer1*, XM_005167299.2; *aak1a*, XM_005167316.2; *ddx51*, NM_001003864.1; *rad 21 L1*, NM_001080050.1; *fkbp1aa*, NM199945.1. GenBank accession numbers of the adjacent genes of medaka *tas1r2a* (NM_001104858.1), *tas1r2b* (NM_001104723.1) and *tas1r2c* (NM_001104724.1) in the figure were as follows: *prdm16*, XM_011476903.1; *tprg1l*, XM_004070640.2; *wrap73*, XM_004070359.2; *tp73*, XM_004070358.2; *rer1*, XM_011476898.1; *gpr153*, XM_011476896.1; *acot7*, XM_011476894.1; *hes2*, XM_004070354.2; *espn*, XM_011476893.1; *arhgef16*, XM_011476892.1; *ybx1*, NM_001104673.1; *ppih*, XM_004070353.2. GenBank accession numbers of the adjacent genes of fugu *tas1r2a* (NM_001105217.1) and *tas1r2b* (NM_001105218.1) in the figure were as follows: *prdm16*, XM_003963257.1; *tprg1l*, XM_003963136.1; *wrap73*, XM_003963258.1; *tp73*, XM_003963138.1; *rer1*, XM_003963139.1; *gpr153*, XM_003963260.1; *acot7*, XM_003963140.1; *hes2*, XM_003963261.1; *espn*, XM_003963263.1; *arhgef16*, XM_003963264.1; *ybx1*, XM_003963141.1; *ppih*, XM_003963144.1

Synteny analysis showed that the *gcT1R2* genes are located on linkage group 21 (Fig. 2). The adjacent genes of *gcT1R2A* and *gcT1R2B* were identical with those of zebrafish *T1R2.1* and *T1R2.2*. Interestingly, the order of adjacent genes of *gcT1R2C-F* was also identical with the order of the same genes in zebrafish without any gene at the corresponding location. In addition, as the different color-marked gene groups shown in Fig. 2, gene translocation might be occurred among the four fished we selected.

**Fig. 3:**
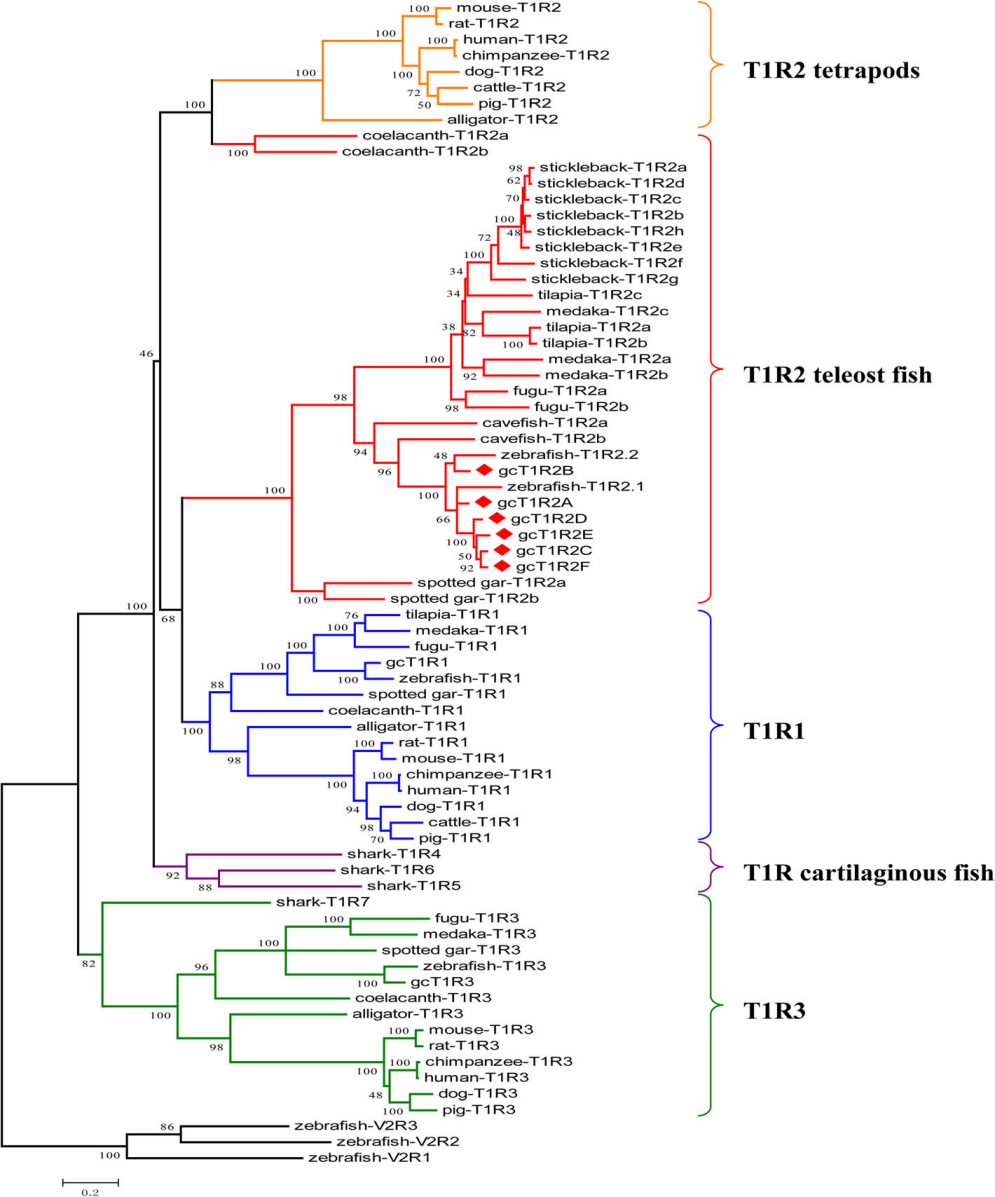
Molecular phylogenetic analysis by maximum likelihood method of T1R2**.** The evolutionary history was inferred by using the Maximum Likelihood method based on the JTT matrix-based model. The tree with the highest log likelihood (− 39,600.8248) is shown. The percentage of trees in which the associated taxa clustered together is shown next to the branches. Initial tree(s) for the heuristic search were obtained automatically by applying Neighbor-Join and BioNJ algorithms to a matrix of pairwise distances estimated using a JTT model, and then selecting the topology with superior log likelihood value. A discrete Gamma distribution was used to model evolutionary rate differences among sites (5 categories (+*G*, parameter = 2.8034)). The rate variation model allowed for some sites to be evolutionarily invariable ([+*I*], 1.3917% sites). The tree is drawn to scale, with branch lengths measured in the number of substitutions per site. The analysis involved 73 amino acid sequences. All positions containing gaps and missing data were eliminated. There were a total of 503 positions in the final dataset. Evolutionary analysis was conducted in MEGA7. The *T1R2s* amino acid sequences of fishes and mammals used are given in the electronic Additional file 3: Table S2

Phylogenetic analysis showed that all fish T1R2s and mammalian T1R2 formed two independent clusters except coelacanth (Fig. 3). The coelacanth T1R2s are more closely related to mammalian T1R2. The teleost T1R2s were more closely related to the tetrapod T1R1s than to the tetrapod T1R2s. In fishes, spotted gar (*Lepisosteus oculatus*) which belongs to *Holostei* formed an independent cluster. For Teleostei, Ostariophysi, containing zebrafish, cavefish (*Astyanax mexicanus*) and grass carp, and Acanthopterygii, containing medaka, fugu, stickleback (*Gasterosteus aculeatus*) and tilapia (*Oreochromis niloticus*) formed two independent clusters. Notably, gcT1R2A and zebrafish T1R2.1 formed one cluster as well as the gcT1R2C-F, gcT1R2B and zebrafish T1R2.2 formed another cluster.

**Fig. 4:**
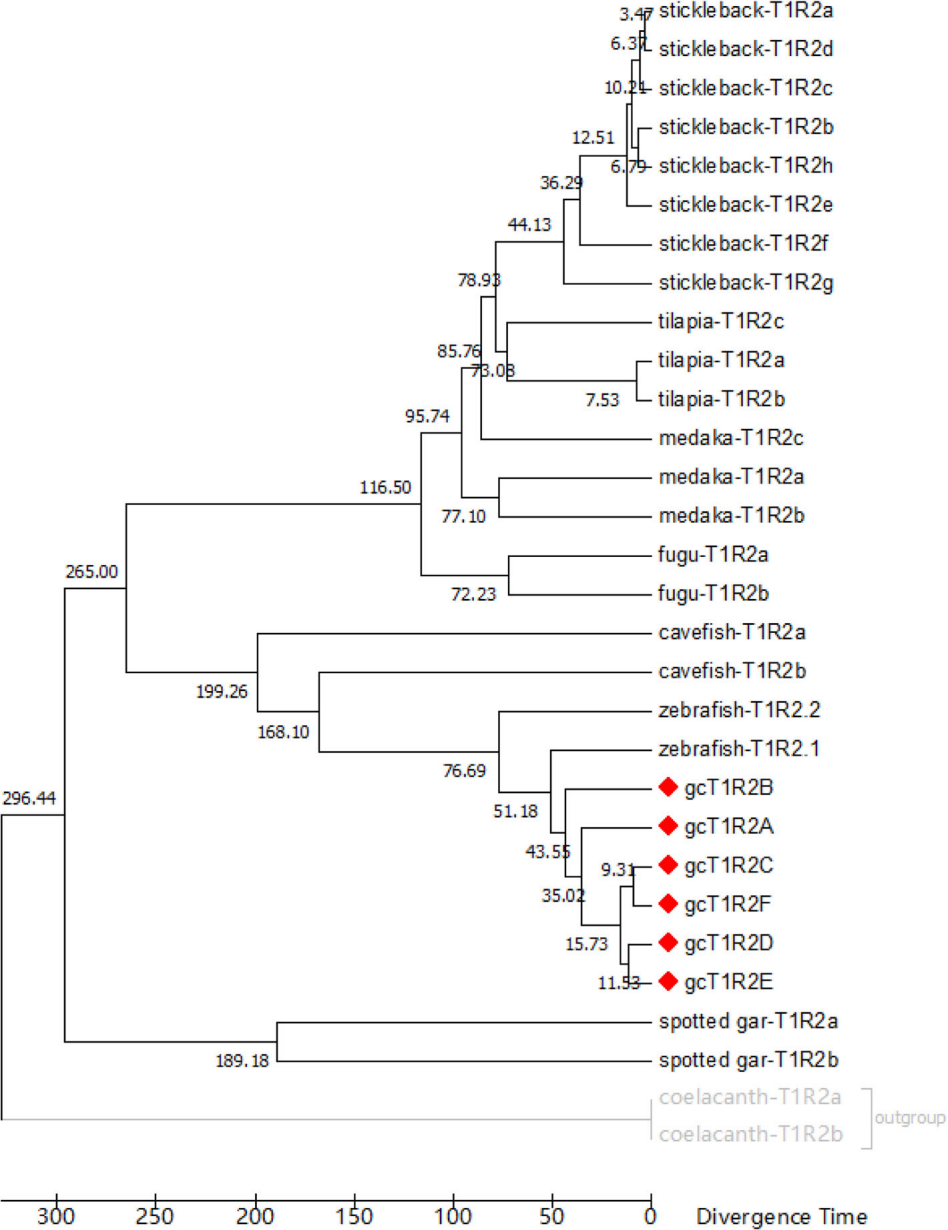
A timetree inferred using the reltime method and the general time reversible model of fish *T1R2* genes. The timetree was computed using 4 calibration constraints. The estimated log likelihood value is − 21,587.6247. A discrete Gamma distribution was used to model evolutionary rate differences among sites (5 categories (+*G*, parameter = 1.6461)). The rate variation model allowed for some sites to be evolutionarily invariable ([+*I*], 9.5713% sites). The analysis involved 30 nucleotide sequences. All positions containing gaps and missing data were eliminated. There were a total of 1353 positions in the final dataset. Evolutionary analysis was conducted in MEGA7. The *T1R2s* nucleotide sequences of fishes used are given in the electronic Additional file 4: Table S3

According to the TimeTree database, *gcT1R2C-F* were formed after the formation of *gcT1R2A* and *gcT1R2B* (Fig. 4). The estimated divergence time between the original formed two *gcT1R2s* and the new formed four *gcT1R2s* was around 34.7 million years ago.

### Tissue distributions of *gcT1R2s*

**Fig. 5:**
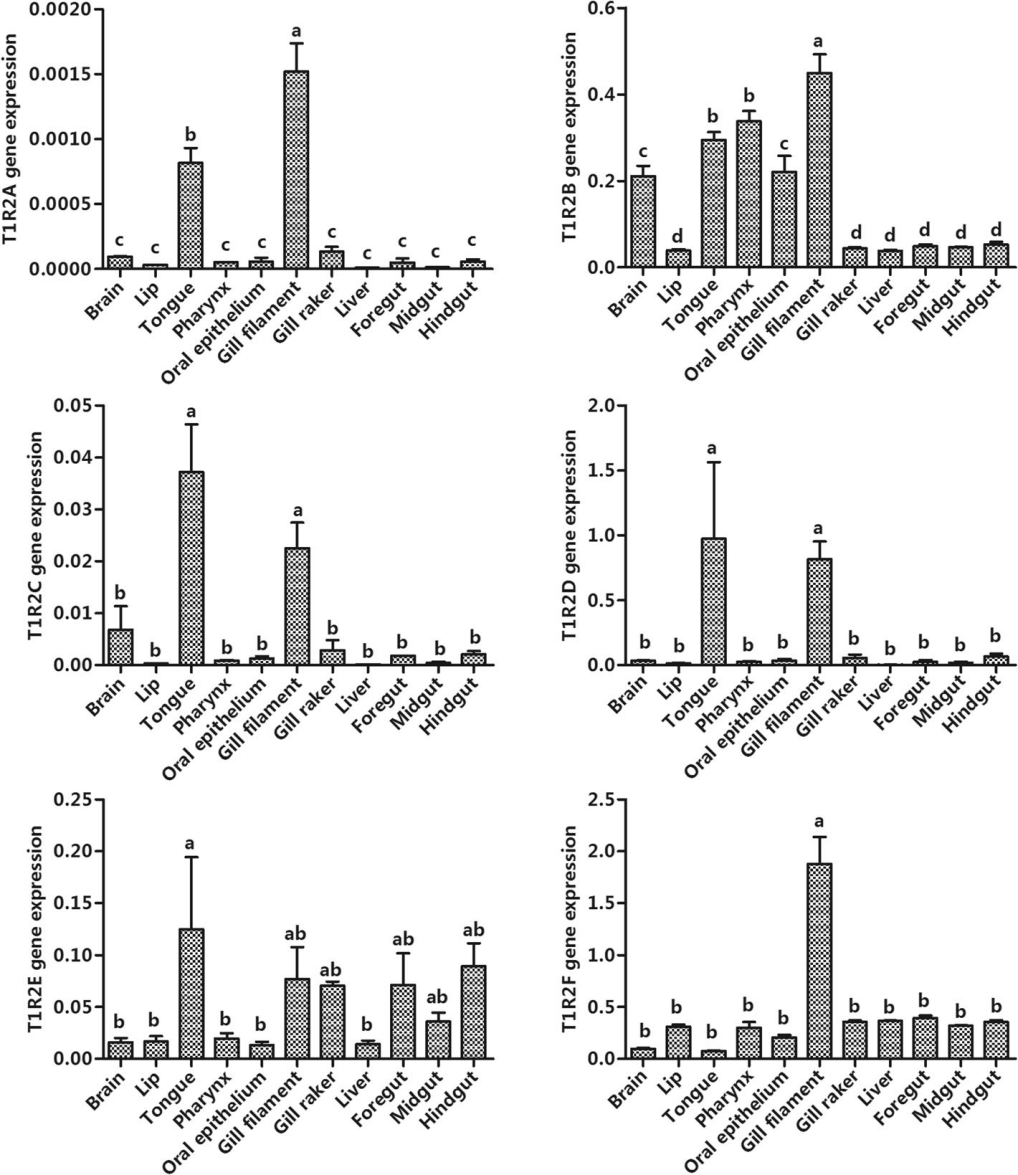
Tissue distributions of *gcT1R2s*. Relative mRNA expression was quantified using real-time PCR and normalized against EF1 as a housekeeping gene. All values represent the mean ± S.E.M. (*n* = 6). Values marked with different lowercase letters are significantly different (one-way ANOVA, *P* < 0.05)

By using geNorm software, the gene with the most stable expression across the experimental conditions was EF1 (Additional file 6: Table S5). The mRNA tissue expression levels of *gcT1R2s* were analyzed by real-time PCR, using EF1 as an internal control (Fig. 5). The gene expression of *gcT1R2A* was the highest in gill filament and followed by tongue. The highest mRNA abundance of *gcT1R2B* was observed in gill filament, followed by tongue and pharynx, and the lower gene expressions were detected in brain and oral epithelium. *gcT1R2C* and *gcT1R2D* were prominently expressed in gill filament and tongue. The gene expression of *gcT1R2E* was the highest in tongue, and abundant in gill filament, gill raker, foregut, midgut, and hindgut. *gcT1R2F* was prominently expressed in the gill filament.

### Response of gcT1R2s to taste substances

**Fig. 6:**
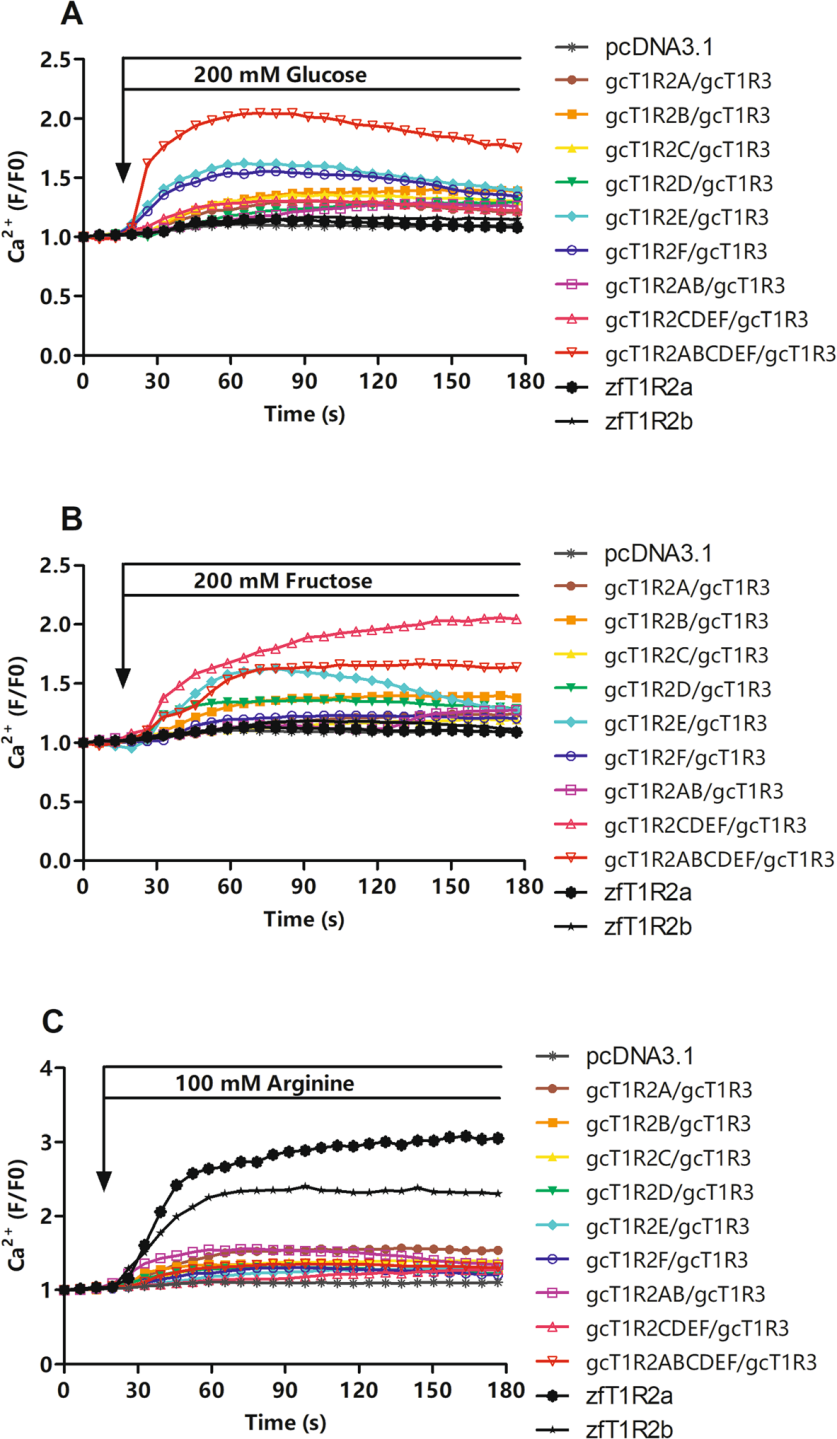
Ca^2+^ changes in fluorescence intensity of 20 single HEK293T cells upon taste substances. HEK293T cells were stimulated with 200 mM glucose (**a**), 200 mM fructose (**b**), and 100 mM arginine (**c**). Images were recorded at 6.54 s intervals up to 183.16 s using 488 nm excitation filter and 516 nm emission filter and analyzed using FV10-ASW 3.1 Viewer software. The backgrounds of the emission intensities were subtracted. Data are expressed as the ratio of the fluorescence intensities of 20 single HEK293T cells per dish and initial intensity (F/F0)

The response curves of gcT1R2s to glucose and fructose were shown in Fig. 6a and b. Each gcT1R2/gcT1R3 could mediate glucose- and fructose-induced intracellular calcium signaling compared with the vehicle (pcDNA3.1 transfected group). Moreover, compared with the response of zfT1R2s/zfT1R3 transfected cells, gcT1R2s/gcT1R3 transfected cells presented higher responses to these two sugars. HEK293T cells transfected with gcT1R2E/gcT1R3 and gcT1R2F/gcT1R3 showed a greater response to glucose than those gcT1R2s/gcT1R3 transfected alone. GcT1R2E/gcT1R3 also mediated more active fructose-induced intracellular calcium signaling. Upon stimulation with glucose and fructose, gcT1R2A-F/gcT1R3 mediated a more intensive and sustained calcium signal transduction than gcT1R2/gcT1R3 alone when transfected into HEK293T cells. Cells co-transfected with six gcT1R2s/gcT1R3 showed a lower response to plant specific fructose than those co-transfected with the new four gcT1R2C-F/gcT1R3.

The response curves of gcT1R2s to arginine were presented in Fig. 6c. Each gcT1R2/gcT1R3 could mediate arginine-induced intracellular calcium signaling compared with the vehicle (pcDNA3.1 transfected group). Moreover, the solo gcT1R2s/gcT1R3 transfected cells presented lower responses to this L-amino acid when compared with the response of zfT1R2s/zfT1R3 transfected cells. No significant differences were observed in the responses of cells to arginine between the solo gcT1R2s/gcT1R3 transfected cells and combined gcT1R2s/gcT1R3 co-transfected cells (included gcT1R2A-B/gcT1R3, gcT1R2C-F/gcT1R3 and gcT1R2A-F/gcT1R3).

### The behavioral experiment of perceiving the sugar in grass carp

**Fig. 7:**
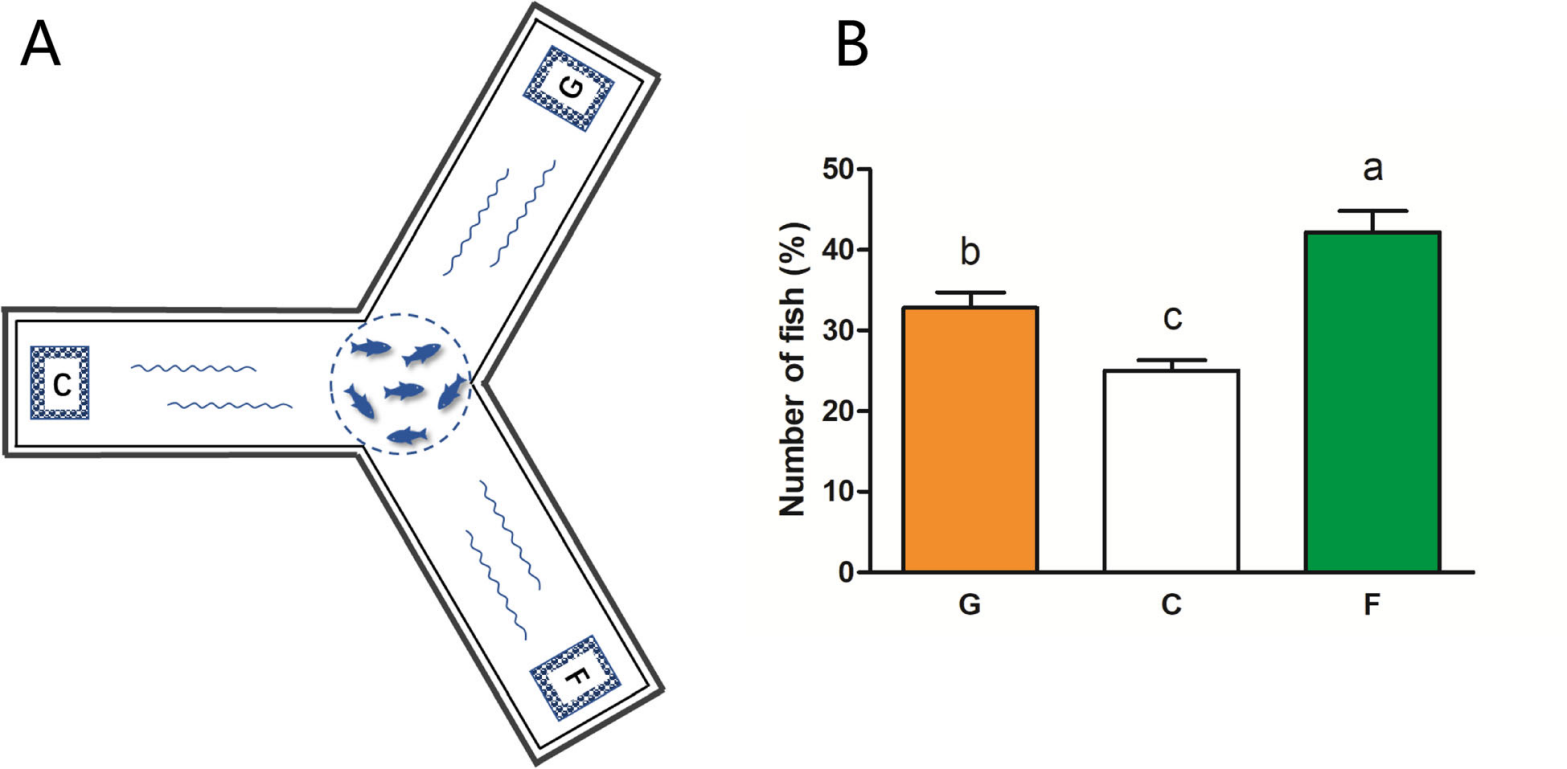
The behavioral experiment of perceiving the sugar in grass carp. **a** Schematic drawing of experimental setup; **b** The ratio of fish chose to different experimental feed placement areas at 0.5 h after the transparent net opening (%). The behavioral experiment was repeated for five times. All values represent the mean ± S.E.M. (*n* = 5). Values marked with different lowercase letters are significantly different (one-way ANOVA, *P* < 0.05)

To examine whether gcT1R2/3 function indeed dictates the taste behavior, we performed one behavioral experiment to confirm the behavioral preference to glucose or fructose in grass carp with a tailor-made Y-maze tank (Fig. 7a). At 0.5 h after transparent net opening in the centre of the Y-maze tank, the ratio of fish chose fructose (F group, agarose and fructose mixed granules) was highest, followed by fish chose glucose (G group, agarose and glucose mixed granules), and the lowest rate of fish was observed in the control group (C group, agarose granules) (Fig. 7b).

### Gene expression of *T1R2s* in grass carp of food habit transition from carnivory to herbivory

**Fig. 8:**
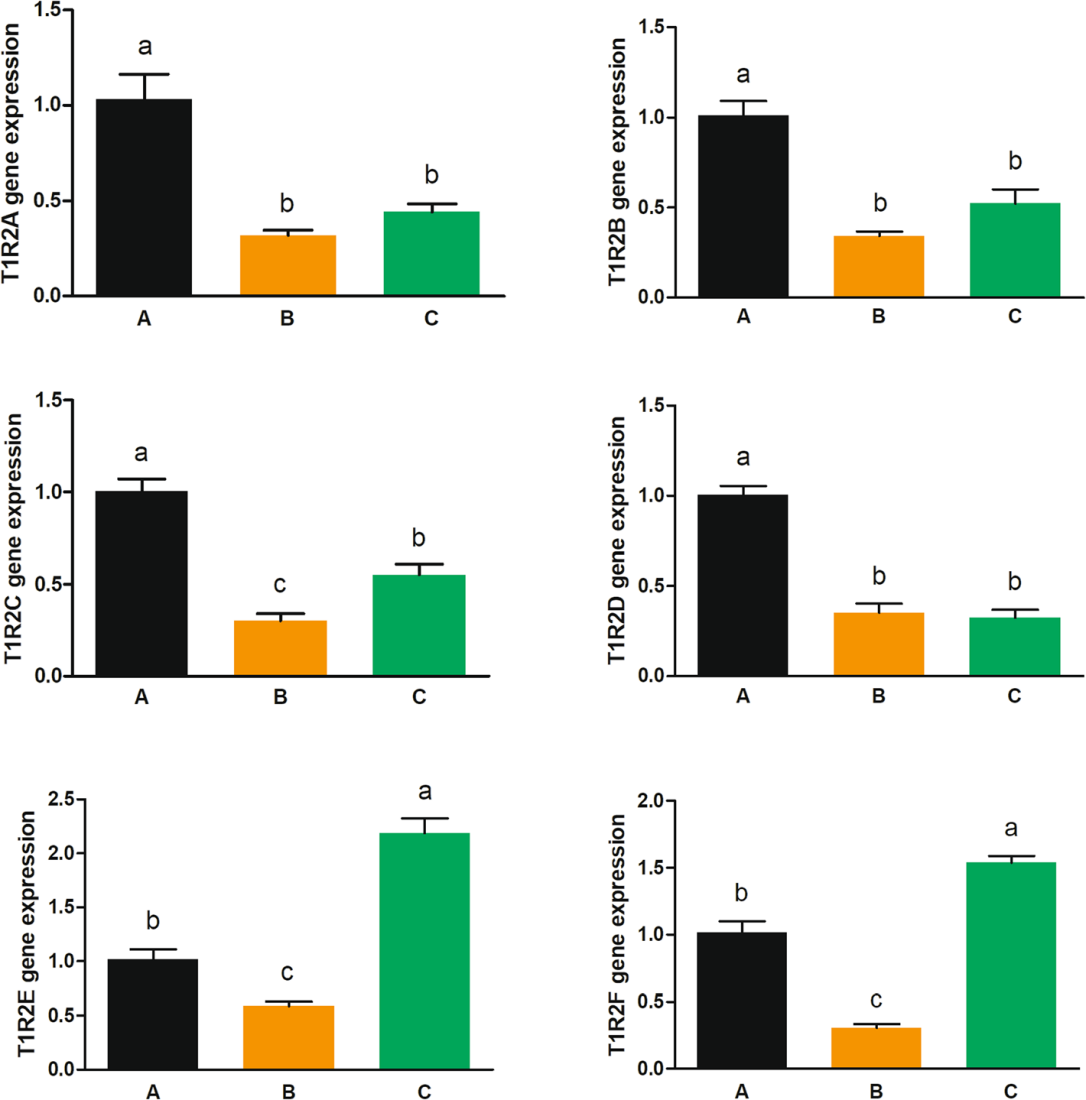
The gene expressions of *gcT1R2s* in the tongue of grass carp transition from carnivory to herbivory. Relative mRNA expression was quantified using real-time PCR and normalized against EF1 as a housekeeping gene. All values represent the mean ± S.E.M. (*n* = 6). Values marked with different lowercase letters are significantly different (one-way ANOVA, *P* < 0.05)

**Fig. 9:**
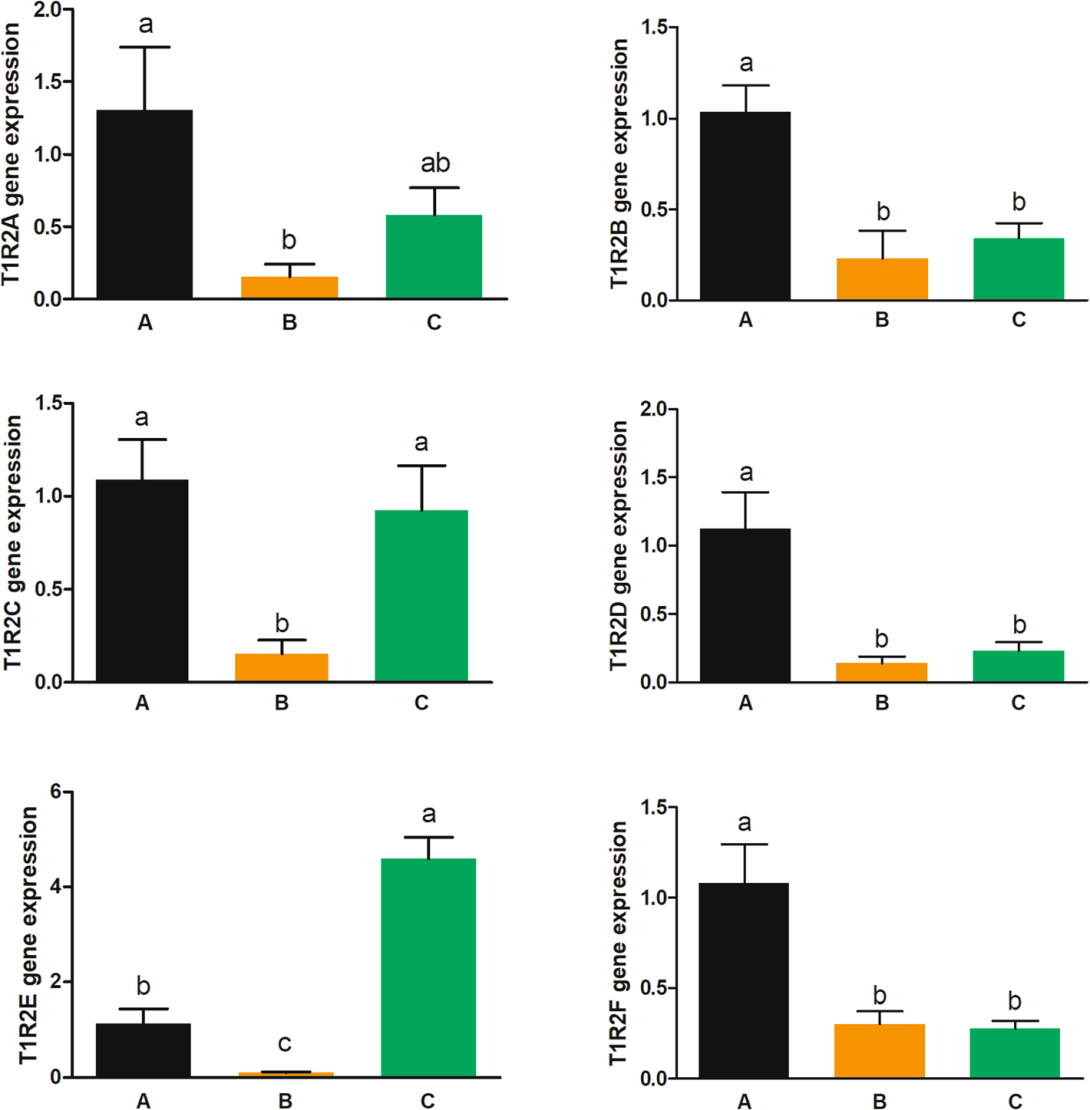
The gene expressions of *gcT1R2s* in the gut of grass carp transition from carnivory to herbivory. Relative mRNA expression was quantified using real-time PCR and normalized against EF1 as a housekeeping gene. All values represent the mean ± S.E.M. (*n* = 6). Values marked with different lowercase letters are significantly different (one-way ANOVA, *P* < 0.05)

Grass carp without food habit transition (fed with chironomid larvae, Group B) had significantly lower gene expressions of *gcT1R2s* in both tongue and gut compared with fish before food habit transition (before the feeding trail, Group A) (Figs. 8 and 9). Fish after food transition (fed with duckweed, Group C) had significantly higher gene expressions of *gcT1R2C*, *gcT1R2E* and *gcT1R2F* in tongue than fish without transition (Groups B) (Fig. 8). And Fish in Group C had significantly higher gene expressions of intestinal *gcT1R2C* and *gcT1R2E* than Groups B (Fig. 9). Moreover, compared with fish before food habit transition (Group A), the gene expressions of *gcT1R2E* and *gcT1R2F* were significantly increased in the tongue of fish after the food habit transition (Group C) from carnivory to herbivory. Parallelly, the gene expression of *gcT1R2E* was significantly enhanced in the gut of fish in Group C compared with Group A.

## Discussion

The genetic basis underlying the formation of food habits in fish is largely unknown [24]. The relationship between the evolution of sweet taste receptor and food habit in fish calls for further investigations. By screening the gene numbers of *T1R2s* in 15 teleost fishes with variant food habits from different orders, we found eight *T1R2* genes and six *T1R2* genes in morphologically and ecologically diverse threespine stickleback and typical herbivorous grass carp, in contrast to two or three *T1R2* genes reported in some carnivorous and omnivorous fish species. Duplication of *T1R2* genes of stickleback, resulting in enhanced perception for substances important for survival and reproduction, has been suggested as an adaptive strategy to varied environment [28]. In the present study, we observed that high *T1R2* duplications in grass carp (six copies) as well as blunt snout bream are related to the vegetarian adaptation through the comparative analysis in cyprinid evolution [29]. Therefore, in addition to previously reported pseudogenization of *T1R2* in mammals and gene loss in birds, adaptive gene expansion in fishes adds a new layer of complexity to the whole evolutionary story of sweet taste.

The *gcT1R2C-F* were evolved from and paralogous to the two original *gcT1R2s* (*gcT1R2A* and *gcT1R2B*) according to evolutionary analyses. The estimated divergence time was around 34.7 million years ago. The gcT1R2A and zebrafish T1R2.1, gcT1R2B and zebrafish T1R2.2 each formed an independent cluster, respectively. The original two gcT1R2s are paralogous to the new formed four gcT1R2s and orthologous genes to two zebrafish T1R2s according to the synteny analysis. Meanwhile, the transposition of genes nearby *T1R2s* among the four selected fishes might accelerate the duplication of *T1R2s* [30].

We found six *gcT1R2s* were all expressed in the taste organs such as tongue and gill. In mammals, *T1R2* and *T1R3* are expressed in glucose sensing cells of the gastrointestinal tract, where they play important roles in nutrient detection, perception and assimilation [31]. In the present study, *gcT1R2s* were expressed in not only taste organs but also intact gut, indicating their functions in glucose detection and perception. In particular, certain expressions of *gcT1R2B*, *gcT1R2E* and *gcT1R2F* genes were detected in brain, suggesting that partial gcT1R2s might be participated in glucose-sensing in brain like the roles in mammals [32].

Signal transduction of gcT1R2s were determined through calcium imaging analysis in HEK293T cells for functional study. Each gcT1R2/gcT1R3 could mediate glucose-, fructose-, and arginine-induced intracellular calcium signaling. Previous studies also reported that sweet taste stimuli elicited an increase in Ca^2+^ concentration in mammalian taste cells [4, 33, 34]. Previous studies have reported that transient receptor potential channel M5 and phospholipase C-beta 2 colocalized in fish taste receptor cells, indicating vertebrates share a common molecular component in taste signal transduction [22, 35–37]. Therefore, glucose and fructose activated the intracellular calcium signaling mediated by each gcT1R2/gcT1R3, indicating all gcT1R2s were functional genes.

A previous study that characterized the ligands for T1Rs in zebrafish and medaka fish has shown that both zebrafish and medaka T1R2s/T1R3 responded to some L-amino acids but not to sugars [38]. In the present study, although gcT1R2s/gcT1R3 responded to arginine were not as strong as zfT1R2s/T1R3, gcT1R2s/gcT1R3 still responded to L-amino acid. However, gcT1R2s/gcT1R3 responded to sugars in the current study, which was different from the known response of fish T1R2/3 to sugars. As far as we know, it is the first cell-based evidence of sugar-sensing receptors in teleost fish. Compared to the previous work, especially the experimental results of zebrafish T1R2s/T1R3, the function of T1R2s in grass carp seemed to be transformed to respond to sugars, especially the grass carp-specific duplicated T1R2s.

In addition, a behavioral experiment was performed to confirm the behavioral preference to glucose or fructose in grass carp with a tailor-made Y-maze tank. At 0.5 h after transparent net opening in the centre of the Y-maze tank, the ratio of fish chose fructose was significantly higher than the ratio of fish chose glucose, suggesting grass carp preferred fructose to glucose under the same circumstance. In in vitro experiment, upon stimulation with glucose and fructose, gcT1R2A-F/gcT1R3 mediated a more intensive and sustained calcium signal transduction than gcT1R2/gcT1R3 alone. Interestingly, plant specific fructose stimulated a more active calcium signaling in cells transfected with gcT1R2C-F/gcT1R3 than gcT1R2A-F/gcT1R3, raising the possibility that change in food environment might be a major selective force shaping the adaptive evolution of the gcT1R2s. Therefore, these results indicated that the gene expansion, especially the formation of new four gcT1R2s, was an adaptive strategy to dietary switch.

As grass carp goes through a transition from carnivory to herbivory during its life cycle, the gene expressions of *gcT1R2s* before and after the food habit transition should be detected to determine its contribution to diet selection. The gene expressions of *gcT1R2s* in both tongue and gut of grass carp without transition (Group B) were significantly decreased compared with the fish before food habit transition (Group A) fed with the same feed, suggesting the glucose-sensing of this fish might change during growth and development. Despite the changed glucose-sensing during development, food habit transition from carnivory to herbivory was accompanied by increased gene expression of certain *gcT1R2s* in both tongue and gut when compared fish after food transition (fed with duckweed, Group C) with fish without transition (fed with chironomid larvae, Groups B). In our previous study, we have declared the food habit transition from carnivory to herbivory in grass carp might be due to enhanced gut growth, increased appetite, resetting of circadian phase and enhanced digestion and metabolism, as well as extensive alternative splicing and novel transcript [25, 26]. Here, we found regulation of *gcT1R2s* expression also drove the food habit transition. Thus, both gene expansion and expression patterns of *gcT1R2s* contributed to food habit transition from carnivory to herbivory during the evolution of grass carp.

## Conclusions

Six sweet taste receptors (*gcT1R2A-F*) were identified in grass carp which exhibits food habit transition from carnivory to herbivory. The four gcT1R2s (*gcT1R2C-F*) have been suggested to be evolved from and paralogous to the two original *gcT1R2s* (*gcT1R2A* and *gcT1R2B*). All gcT1R2s were expressed in taste organs and mediated glucose-, fructose- or arginine-induced intracellular calcium signaling, revealing they were functional. In addition, grass carp was performed to prefer fructose to glucose under a behavioral experiment. Parallelly, compared with gcT1R2A-F/gcT1R3 co-transfected cells, gcT1R2C-F/gcT1R3 co-transfected cells showed a higher response to plant-specific fructose, indicating the gene expansion, especially the formation of these new four gcT1R2s, was an adaptive strategy to dietary switch. Moreover, the regulation of *gcT1R2s* expression drove the food habit transition of grass carp during development. Collectively, our studies provided some evolutional and physiological clues for the formation of herbivory in grass carp.

## Methods

### Data mining and DNA sequencing

A 0.9-Gb draft genome of a gynogenetic female grass carp adult and a 1.07-Gb genome of a wild male adult are available at the official National Center for Gene Research website (http://www.ncgr.ac.cn/grasscarp/). TBLASTN searches were conducted with *E*-value 10^− 10^ against the genomic data using the available *T1R2* coding sequence (CDS) of zebrafish *Danio rerio*, medaka *Oryzias latipes*, and fugu *Takifugu rubripes*. Each region of BLAST similarity was extended 5–10 kb in 5′ and 3′ directions to establish a detailed prediction of CDS. The screened sequences were estimated based on the profile hidden Markov model (HMM)-based gene prediction with the program WISE2 [39]. The exon-intron junctions were determined by comparing the genomic sequence with the cDNA sequence using SPIDEY. Then, the cDNA of grass carp tongue was used to verify the obtained sequences. The polymerase chain reaction (PCR) was conducted on Biometra Thermocyclers (Biometra, Germany) using Phanta® Super-Fidelity DNA Polymerase (Vazyme Biotech, Jiangsu, China) with the designed primers (electronic Additional file 2: Table S1). The sequences obtained from the genomic database were named as *gcT1R2* genes.

The gene number of sweet taste receptors in teleost fishes was also investigated. By screening from GenBank and previous studies, we obtained the available sequences of *T1R2* genes in 15 species of fishes with variant food habits from different orders.

### Synteny analysis of *T1R2* genes

To determine whether *gcT1R2* genes are orthologous to other fish species, we performed a synteny analysis by screening *T1R2* flanking genes of zebrafish, medaka and fugu through Genome Data Viewer (GDV).

### Alignment and phylogenetic analysis

The T1R2s amino acid sequences of fishes and mammals used in this study are available in NCBI and Ensembl genome browser (electronic Additional file 3: Table S2). Amino acid sequence alignments were performed by ClustalW2.

The evolutionary history was inferred by using the Maximum Likelihood method based on the JTT matrix-based model [40]. The zebrafish V2Rs were selected as the outgroup. Evolutionary analysis was conducted in MEGA7 [41].

### Timetree analysis

The *T1R2s* nucleotide sequences of fishes selected are available in NCBI and Ensembl genome browser (electronic Additional file 4: Table S3). Nucleotide sequence alignments were performed by ClustalW2.

A timetree inferred using the Reltime method [42] and the General Time Reversible model [43]. The coelacanth *T1R2s* were selected as the outgroup. Evolutionary analysis was conducted in MEGA7 [41].

### *T1R2s* expressions in various tissues

Grass carp was obtained from the Fish Center of Xiantao, Hubei, China. The fish was fed to apparent satiation with a commercial diet (32.0% protein; 9.0% fat; 6.9% moisture; 7.6% ash) twice a day at 08:00 and 16:00 (Beijing time) under a standard laboratory condition. After the 2-week acclimation, six large grass carp (500.9 ± 57.6 g) used for tissue distributions of *gcT1R2s* were deeply anesthetized with MS222 (200 mg L^− 1^). The brain, lip, tongue, pharynx, oral epithelium, gill filament, gill raker, liver, foregut, midgut, and hindgut samples were collected. RNA extraction and cDNA transcription were performed with Trizol reagent (Takara, Japan) and PrimeScript™ RT reagent Kit with gDNA Eraser (Takara) according to manufacturer’s protocols.

The primer sets for *T1R2s* were designed (electronic Additional file 2: Table S1). A set of six housekeeping genes (*β-actin*, *RPL13A*, *EF1*, *TUA*, and *GAPDH*) were selected from the transcriptome assemblies [44] to test their transcription stability for tissue panel. GeNorm software was used to compute the expression stability values (M) for each gene where a lower M value corresponds to more stable gene expression.

Real-time PCR assays were carried out on a quantitative thermal cycler (MyiQ™ 2 Two-Color Real-Time PCR Detection System, BIO-RAD, USA) using AceQ® qPCR SYBR® Green Master Mix (Vazyme Biotech) with the designed primers (electronic Additional file 2: Table S1). The PCR parameters were 95 °C for 3 min followed by 40 cycles at 95 °C for 10 s, annealing temperature for 30 s, and a melt curve step. Primer PCR efficiencies of the genes ranged from 97.8 to 102.5%. Gene expression levels were quantified relative to the expression of housekeeping genes using the optimized comparative Ct (2^-ΔΔCt^) value method [45].

### Preparation of recombinant expressional vectors, cell culture and calcium imaging

The complete coding sequences of two *zfT1R2s* (zebrafish *T1R2a* and *T1R2b*) and *zfT1R3*, and six *gcT1R2s* and *gcT1R3* were subcloned into the pcDNA3.1 expression vector (Invitrogen, Carlsbad, CA) used ClonExpress™ II (Vazyme Biotech), respectively. HEK293T cells were cultured in Dulbecco’s modified Eagle’s medium (DMEM) (Life Technologies, Carlsbad, CA, USA) supplemented with 10% fetal bovine serum (Sigma-Aldrich, Saint Louis, MO) at 37 °C in 5% CO_2_. The cells were plated at a density of 1 × 10^6^ cells per 20-mm glass bottom cell culture dish the day before the experiment. After 14 h, the cells were transiently transfected 24 h before the experiment with the gcT1R2s/gcT1R3 recombinant expressional plasmids by using Lipofectamine 2000 reagent (Invitrogen). For nutrient starvation experiments, HEK293T cells were placed in phenol-free/glucose-free DMEM (Life Technologies) for 3 h. The experiments were set up three parallel 12 groups: the control group transfected with pcDNA3.1 only; the next six groups co-transfected with sole gcT1R2s and gcT1R3 (gcT1R2/gcT1R3); the 8th group co-transfected with gcT1R2A, gcT1R2B and gcT1R3 (gcT1R2A-B/gcT1R3); the 9th group co-transfected with gcT1R2C, gcT1R2D, gcT1R2E, gcT1R2F and gcT1R3 (gcT1R2C-F/gcT1R3); the 10th group co-transfected with all six gcT1R2s and gcT1R3 (gcT1R2A-F/gcT1R3); the last two groups transfected with zfT1R2a/zfT1R3 and zfT1R2b/zfT1R3.

After 3 h nutrient starvation experiments, cells were washed three times with Dulbecco’s phosphate-buffered saline without calcium and magnesium (DPBS) (HyClone Lab, Logan, UT). Cells were loaded with 4 μM the calcium-bound Fluo-4 dye (Invitrogen) diluted in DPBS for 30 min at 37 °C in 5% CO_2_ and then washed three times with DPBS and incubated for an additional 30 min at 37 °C. Dishes were placed on the stage of an inverted confocal microscope (FluoView FV1000; Olympus, Tokyo, Japan). The dishes were perfused with 200 mM glucose, 200 mM fructose and 100 mM arginine (Biosharp, Hefei, China) at a rate of 2 mL/min after the first 3 pictures were taken. Baseline was established for at least 15 s before stimulation. Three series of 12 groups cell dishes were treated with 200 mM glucose, 200 mM fructose and 100 mM arginine diluted in DPBS separately. Images were recorded at 6.54 s intervals up to 183.16 s using 488 nm excitation filter and 516 nm emission filter and analyzed using FV10-ASW 3.1 Viewer software. The backgrounds of the emission intensities were subtracted. Data are expressed as the ratio of the fluorescence intensities of 20 single HEK293T cells per dish and initial intensity (F/F0).

### The behavioral experiment of perceiving the sugar in grass carp

Before the behavioral experiment, three experimental feeds were prepared by hand, which were small agarose granules (namely C group), agarose and glucose mixed granules (namely G group), and agarose and fructose mixed granules (namely F group), respectively. Grass carp (14.36 ± 0.15 g) were obtained from the fishing ground (Wuhan, China) and placed in a 1000-L tank 1 day prior to the start of experimentation. On the training day, fish was placed at the centre of a tailor-made Y-maze tank with a transparent net to prevent escape (seen in Fig. 7a). Then, the three experimental feeds were placed at the end of different channels separately. At 0.5 h after transparent net opening, the ratio of fish chose to different experimental feed placement areas were counted.

### *T1R2s* gene expression analysis of the food habit transition from carnivory to herbivory in grass carp

Fish and samples were prepared according to our previous experiments of He et al. [25]. The fish embryos were obtained from Wuhan Academy of Agricultural Science and Technology (Wuhan, Hubei Province, China). Grass carp larvae as raised in tanks and fed with chironomid larvae (*Chironomus tentans*). At days 46 post-hatch (dph) (body weight 0.39 ± 0.05 g, body length 28.05 ± 0.99 mm), fish was randomly selected for sample collection as fish before food habit transition (Group A). The rest of the fish was randomly divided into two groups (*n* = 1000 for each group) fed with either chironomid larvae as fish without transition (Group B) or duckweed (*Lemna minor*) as fish after food habit transition to herbivory (Group C). An excess of food was offered 24 h a day and fed for 70 days. At 116 dph (body weight and body length for Group B was 2.97 ± 0.3 g and 53.96 ± 1.80 mm, respectively; those for Group C was 7.34 ± 1.43 g and 72.78 ± 6.15 mm, respectively), 6 fish were randomly selected from the groups for sample collection. The tongue and gut of grass carp were collected and then frozen in liquid nitrogen and stored at − 80 °C for RNA. Total RNA was isolated, and cDNA synthesized as mentioned above.

To detect the gene expressions of *T1R2s* in grass carp of food habit transition from carnivory to herbivory, real-time PCR assays were carried out as mentioned above.

### Statistical analysis

All data were presented as mean ± S.E.M (standard error of the mean). The normality of data was assessed by using SPSS software with the Shapiro-Wilk test. All data were subjected to one-way analysis of variance (one-way ANOVA) using SPSS 17.0 software. Differences between the means were tested by Duncan’s multiple range test after homogeneity of variances was checked. Statistical significance was determined at the 5% level.

## Supplementary information

**Additional file 1: Dataset S1.** The genome sequences of the grass carp sweet taste receptor genes. Exon region is uppercase. Intron region is lowercase. The genome sequences of the grass carp sweet taste receptor genes are provided. Exon region is uppercase. Intron region is lowercase.

**Additional file 2: Table S1.** Primer sequences. The primer sets for T1R2s sequencing and functional studies.

**Additional file 3: Table S2.** Amino acid sequence alignments of genes for phylogenetic analysis. Amino acid sequence alignments of those three genes of different species.

**Additional file 4: Table S3.** Gene accession number for Timetree. The T1R2s nucleotide sequences of fishes selected are provided.

**Additional file 5: Table S4.** Amino acid sequence identities of T1R2 of various vertebrates. Amino acid sequence identities of T1R2 of various vertebrates are provided.

**Additional file 6: Table S5.** Expression of candidate reference genes analyzed for the tissue panel using geNorm. GeNorm software was used to compute the expression stability values (M) for each gene where a lower M value corresponds to more stable gene expression.

## Data Availability

The datasets supporting this article have been uploaded as part of the supplementary material.

## Abbreviations

CDS: Coding sequence
DMEM: Dulbecco’s phosphate-buffered saline without calcium and magnesium
EF1: Elongation factor 1
GAPDH: Glyceraldehyde-3-phosphate dehydrogenase
GPCRs: G protein-coupled receptors
HMM: Hidden Markov model
PCR: Polymerase chain reaction
RPL13A: Ribosomal protein L13a
T1R: Taste receptor type 1
T1R2: Taste receptor type 1 member 2
TUA: Tubulin alpha 1
β-actin: Actin isoform β
