# Supplementary material for: Expansion of sweet taste receptor genes in grass carp (Ctenopharyngodon idellus) coincided with vegetarian adaptation
Source: BMC Evol Biol. 2020 Feb 11;20:25. doi: 10.1186/s12862-020-1590-1 (PMC7014666; doi:10.1186/s12862-020-1590-1)
Supplement: Supplementary file 1 — Additional file 1: Dataset S1. The genome sequences of the grass carp sweet taste receptor genes. Exon region is uppercase. Intron region is lowercase. The genome sequences of the grass carp sweet taste receptor genes are provided. Exon region is uppercase. Intron region is lowercase. [file 12862_2020_1590_MOESM1_ESM.docx]

**Dataset S1.** The genome sequences of the grass carp sweet taste receptor genes. Exon region is uppercase. Intron region is lowercase.

>gcT1R2A

gatggtgtaatgtgacacttgttggatgacaaatctggattattgttatatgcaaaagtatttaaattttttctgcaggaactgggttaagcaaatttaatgtaaatgtaaaatgtatgcgaataaccttgctgatttaaggtcactgtttccctaattcagtagcagtggatttcctgaaacaacagatagtggtgctgtttttaccgtagttcattggaaatccatgaatttaggcatcaaccaggaaaggcattaactatttattttttggttgcactttatttttcagtacagtactgtacttacatgtacttacagtgtacttacccaagaaaatagtgggtaatataaggtaactacaggggttaaggttaggtttaggggtaggttcagggtcagtagttattatccagttattttaattactgtaataagtacattgtatgaacatgggaaacggaactgtaaaataaagtgctaccccatttttggttacacttcattttaaggtgtctttgttacactgtaataatacatttaagtattgagtaatattaagtaactacatgtacttactatatgattaggattagggtttagttttgtttagggttagttgcatgtaattatgcataatttataattattactatagtaactaaagtaacgtatgtaacaatgacactgtaaaataataccccatttttttatatacattcaaaatgtgatgtaacaacacagtaatcacctcaacagaatttaatttgtaaatttatgtatgaggagacatttgtggtgtaatgctctacttaataactgattgacatatgctttgtgagtttagatatggaaataggcatttgtatcagtgaatatagtggtttaagcaaatagaaatgtgttttaagcaaattaagtgtagtatatcagccggatttctcttaaacggccagccactacacagccctgtttcaagattaacggtccatcataaaccATGTTTCACAGTAGCATTTACGTTTTCCTTTTGGGCTTTAGTAATTGTTTCTTTTTCAAAGTTTCTTGGGCAACATCTGAGTTCAGCTTGGAAGGAGATTACTTATTAGGTGGCCTTTTTACTTTACATGAAATTGACCAGGTAGAACCAGTTTTCACCCCCGAGACCACTGAATGTTTCAGgtaagcaaggaccttttatccgtctagaattatcattacgcaataatgtaagtaataatgtaagcagcatttcacatttctaaactgaattttcaatgacatgctattaactacctcactgatgttttcaagGCACAGTTCCTCAAAATCTGGCTATCAGATGTTGCAAGTAATGAGGTTTGCCGTTGAGGAGATTAACAACTCCACCACTCTTCTGCCCAATGTTTCTCTGGGCTATGAAATTTTTGACCATTGTTCTAATACAAAGAATTTCCGTTCAGTCTTAAGTTTTATCTCAAATAACGGATCAATAAAACCTAAAGAAAAACTCAACAACTATCAGCCTAAAGTGATTGCTTTAACAGGGCCATATGGAAGCACAAGAACTATTACTATTGCACCACTGATCACAATGGACCTTATACCATTGgtaaatcttaaacagtttacataaaaaaaaaatacgacttcagaactaccaagttacaataatgctaaattattttttataatatagtgttttcttcactttgaaagGTGAATTATGGAGCTTCTAGCTATGCGTTAAGCAATAAACTTCAGTATCCCTCTTTTGTAAGAACAGTTCCCAGCAACAAAGACCTGATAGAGATGATTATTCACATCATACGGTGGTTTGGATGGAACTGGGTTGCCTTTCTTGGTAGCCAAGACAATTACAGTTCAGACGGACTAAAGCTGTTTAACAAGTACATCAACAATACTGGCATTTGTTTGGCCTATCAAGAGGGTCTAAGCCTAAACGCAAACTACAGTCTAACACTTAAAAAGATTGATATGCTCAAGATCAATGTCATTGTGGTTTTTGCTGTACCACAATATGCAATCAACATAATCAAAACAGCCATAGTAGAGAACATACGAGACAAAGTATGGATTGCAAGTGAAACGTGGGCTATGAATCAACAACTTCCAAGAGAGCCAGGAATTGGGAAAATTGGTACAGTCATTGGCATTACGGAGAGATTGCTGTCATTACCTGGATTTGATGAATTCATCTATAAAGAGAGGGGAACAACTGATGTTAATTACAAAGCTGAGAGTGAAGTCCAGGGTAAGAGTAAGACATGTAATCAAGTTTGCGATTACTGCACATTGTTGACTGCAGAGGAGATTATAAATGAGAATCCCTCATTCTCCTTTGCTATCTATGCTGCCATATATACTGTAGCTCATGCATTACATAAGATTCTGCAGTGCGACATGAATGAATGCCACAAAAACAGAATGGCTAAGCCATATATGgtaaattaatttatttgaatccatgaattactgtatattcattattcactattttaatagcaatattttaattaattaattaaataattaacaatgtttatattttcaggaaattgcatattatataaattatattgtattattgttttattgtttgttttatttaataattatacaataactttaatgttatagtaatattattacaaaatatttttcttgtttatttgaacacacttcacaacagCTTCTGGGAGAAATGAAAAAGTTGGATTTTCCACTCAATGGCCGTCAGGTGAAATACGATGATAATTATGATCCAACCATCAGTTACGCAGTTGTGCTTTGGCACACTGATGTGAATTCTCCACAGTTTGAGATGGTGGGCACATATGATACATATCCAGAAGTTACTTTTACCATGGACAACTCTCTTTTGCCCTGGAGTAATAATAATTCTgtaagccttatttatttatttatttattggttggttggttggtgaaccttatctaaacatttagctttgtagaaaacattttctcaatcagttatacatatttctccaaacaaaagtcaatacgctcaaatcaaataatacaaccatctagcaaattcacactattttttgcagctaacacatgaaaagcaaatactgaagtcaaaactactgaactgttctctattgattacatatttatacacaactatactattgaaaatgtgcatttattatacagttgacacaatggagatcttttttccatgtgtttatcttgagctatggatctaaagaaaggacggtgtgaattcacttgaattcatgcctggcatgcatatgcctcaatatctctgcaggcctcttctgttgcttggagaagatttactttgacatagaggttgtggttgaacatcctctatctccaggagaaatcccacaaatggattcagaaatggagaatatagtggaagcagcagcatcacaaaatgtaggtgattttgtaacccacaatgaatggtgaaattggacattgcctaaccccaagacaaacactgaccaatgacattccttctttttttgtttttgttgactatggaatagaaccatctattagagtggctctcatctcatcttgggcactcttgaaagaattttgtaaatctcctttcctcttcacccattccacctttagcctactcttcctcagcccactctacctattattatgcattattatctattctatatgcactactatatgcacttcttttctcttatccccccccccntggtctcttatgcaaggcaaaggcatgatagttgtgaacaattacttactcatgcagaagatatcaagccatgccatttgtataaagaagatggctttatttaagttgtctgttaactgttcagatctgataaatttgtgcatagtgtttaagggaattaattatgtaatttgtattctgtaatttgcagtccatttctggaaacatagcgttcaggctgtgttaattgtttgagaacactgatcttagtttggagaaatgtgtaaaatcgatagagaaaattttgatctttaataacacattttcccattctttaaagGTTCCTTTCTCAAACTGCTCTGTTGAATGTAAGGAGGGATTTTCAAGGCAACCTGAAGGTTTTCATAGTTGCTGTTTTACATGTAAAAAATGCCCGCGTAACAGCTATGTGGATTATTATCgtaagtactttatgtgccggtcaataaaaccttgatatttaagcttcttcagtccattctatcatcacaatagttttggaattagggaactttttgaaatgtgatttgaagaaaaaatattttcctacatccagttactactattatggcttttggcaaaatggtattactaaacattcaaattattttactgtttctcagGGGATCCCTATACCTGTTTTCCATGTGCAGAGAGTGAATGGTCTGATGAGGGCAGCACAACATGTAAGAGACGTTCTGTTGTCTATCTTCAGTTCACAGAAATCTCCTCCATCGCTGTGATGGTTTCTGCCACATGCCTTATTATTCTCCTTTTTGCCATATTTTTCCTTTTCACCTATAATTACAACACACCAGTGGTTAGGTCTGCTGGTGGTAGCATGTGTCTTCTTATGTTGATATGTTTGATAATGTCTAGCATAAGTGTGTTCTTCTTTTTTGGACAACCCACATCTGTACATTGCCTTCTGAGAAATGCCATATTCGCATTTTTCTTCACTGTCTGTCTTTCTTGTTTGACTGTCCGTTCCTTTCAAATTATTTGTGTTTTTAAAATGGCTGCTCAGTTCCCTAAGGTGCACAGCCTTTGGGTAAAGCACAATGGACAGTGGCTCTTCATTGCATTTTCTTCGGCTATTCATTTAATTTCTTGTGTGATATGGATGACTGCTTCTCCTGACAAACCCATCGCTGACTCATGGACTTTTAAAGATCAAATTATGCTCATCTGTGAAATGGGGAACACTATAACCTTCACCATAGTAGTGTTTATAAGTTGGTTTCTTGGTTTCCTCTGTCTTCTGTTTTCTTACATGGGAAGAGATCTGCCAAAAAATTACAATGAGGCCAAATCAATAACTTTCAGTCTAATTTTGTACTACCTGAGTTGGATTGTATACTTCACAGCATACCTCTCTGTCAAAAGCAAGTACATCATGCTTTTGAATGCAATGGCCCAGATATCTAGTATATATGGAATTCTCTTCAGCTATTTCATACCAAAATCTTACATTATAATATTCCAACCTCAAAAGAACACTGCTACATACTTTCAAACATCTATTCAGAATTACACCCAGACCATTAGTAGGACTTAGactacaacgattcaaagttgcagataaagtcatccacaagggaattgcattagatggaggatgattgtgtttttaaagcacagttgtggatatttgacagataaatacatttggcattttagtaagcattccctctctacataccttgtaaactttggcagtaactttagtgtatgtaggaacactgcatgtatgtggttaaattcattgctcaaggtcacagacctcagtaactctctaatccacaaaaccctctacttggttgtgaaccctcttaaactgtaagctttagatccttcactactatgttaccacaatcttaaataaacacataagaaaagtaaagaaggaagatacattctggggttcattctgcactatatcacatgtcatttatggaaaaaaaagagcagtttaaatatttataaagctgttctttacatgctagcatttacacttaagtgttaatgtaaacacaaatgcttgctttttacatgtaataaagaacattttcaaaacttccataactcagtgtctaaataatcaatatagatttgtgaggtttggattgtcaatcgtgtatctttgatgtcatactgaccatctattcacattcgtaacaatatatgtatgtatataggcatatatatatatatatatatatatatattgttacgaatgtgaatagatggtcaatagtgaaggtgtagtgaaggtggactgttgtaaggtaggcttggtcgttgccatggagtgtcggtaaggacacagagacaggaaccccagttaaataattttaatgtcaatcaagtttaaatagtttcatgggtatgggtggtcctttgcatatgtggctaaaacaaatgaaaaattgggtaacactttttaataactttcattaataaatcattaataaatcattaacaattattatgtaatgcttaacagatcattagttaattcatattcatatagctagaaacatgagataatgtgcttg

>gcT1R2B

gaacttccctgacatacactgctgccttattttgtgctttttgcttgtgagtcttgtgagtttattttaccagttttattatacctttatatctccttgtgttgcatatgagtccacttgctgtctgcctttgccagtagctcaggggtaccttgtctgagaaactggggttcaagcacttgtcaaaaaaccttactagttacagagctaatttatgctctaaacgaagaaatattgtgaatgatattattaatccaactgtaaaagccagctgaaactttgcattattgcgagcagtttgttttaggagagttcgagttcgaatagcgaaattcgaaaagaatttgtgtgtgcgcgcgtgtgtgtgtgtgcgcgcgcgcgtgtgtttttgggacatatcaggacacaaatttgtataatgacatgggtatgacataggtattacaaggagagggtgccttactttttttccatatattttttaaaattctttccggtaacactttagtataggaaacacatataaactattaactatgacttttccctcaataaactcttaatttactgcttattaatagttagtaaggcagttgttaagtttaggtattgggtaggattaagaatgtagaataaggtaatgcagaataaggcattaataagtgcttaataagtactagtaaataaccaatattctagtaatatgcatgctaataagcaactagttaaaaaaccctaaaataaagtgttaccttctgtgtatttaaagtgatattttgcatctgttgtttgtgatgtggtgtaatgctctacttaataaccgatggatgtaaacttttttttgagtttaggtatggaaattgtactttgtatcagtgaatacagtggtttaagcaaattgaattgtgttgtaagcaaattccactgttgtatatgaacctgacttctattgcttgccactgcagattctagtttcacaaccaacggtctaccgtaaaccATGCTTCTGAGTAGCATTTACTCTTTCCTTTTGGCCTTTATTAATTGTTTCTCTTTCAAAGTTTCTTGCTTAGAATCTGAGTTCAGCTTGAAAGGAGATTACTTATTGGGTGGCCTTTTTGCTTTACATGAAGTTCAACATGCAACACCTCTGTTCTCCCCAGAAGCCATTGAATGTTCCAGgtaagaaaacacttttttgcagcagcagcatttcttcaacatttaatattcaatataacaaagattcaattaagtgcaattcacttcatcactattgtgttctagATACAGTTTCTCAAAATCTGGCTATCAGATGTTCCAAGTGATGAGGTTTGCTGTTGAGGAAATTAATAACTCCACCACTCTTCTGCCCAATGTTTCTCTGGGCTATGAAATTTTTGACCATTGTTCTGACACAAAGAATTTCGCTTCAGTCTTCAGTTTTATCTCAAAAAATGGATCGATAAAACCCAAAGAAAAACTCAACAACTATCAGCCTAAAGTGATTGCTTTAACAGGGCCATATGGAAGCACAAGAACTATTACTATTGCTCCACTGATCACAATGGACCTTATACCAATGgtaaatagtttttttccccttttaatttttataaagtagtactacacataacacataatactgttttcctcttcttttaaagGTGAATTATGGAGCTACCAGCTTTGCATTAAGTAATAAACTTCAGTACCCCTCTTTTCTAAGAACAGTGCCAAGCAACAAAGACCTGATAGAAATGATTATTCGCATTATACAGTGGTTTGGATGGAACTGGGTTGCCTTTCTTGGTAGCCAAGATGATTACAGTGAAGATGGCCTAAGACTATTTAGCAAGTATATACAAAATACTGGAATTTGTTTGGCCTATCAAGAGGCTCTAAGTCAAAGGGCAAACTACACTCTAACACTTAAAAAGATTGATATGCTTAACATCAATGTCATTGTTGTTTTTGCTGTGCCACAATATGCAAGCAGCATTATCAAAGCAGCCATAGCTAACAACATCCAAAACAAAGTATGGATTGCAAGTGAAACATGGGCTATGAATCAACAGCTTCCAAGAGAGCCAGGAATTGGGAAAATTGGAACAGTTATTGGTATTACAGAAAGATTGTTGTCTTTGCCCGGATTTGATGAATTCATCTATAAAGAGAGGGGAACAACTGATGTTAACCATAATGATAGTGCTGATAGTGAAGTCAAGAGTAATAGTAAGACATGTAATCAAGATTGTGATTGCTGCACATTGTTGACCGCAGAGGAGATTATAAATGAAAATCCCACATTCTCCTATGCCATCTATGCTGCCATATATACCATAGCTCATGCATTACATAAGGCTCTGCAGTGCGACATGAATGAATGCTGCAAAAACGCAACAGTTAAGCCATACATGgtgagtgaatgcatcataagataatgaaattttaaaggaaaagtaatattaattcttatattagcagcattctttatatgaattgggacatttttaccacactttaaaacagCTTCTAGGACAAATCAAGAAGTTGGACTTCCCACTTAACGGCCGTCAGGTGAAATATGATGATAATGGTGATCCAACTGTCAGTTTTTCAGTCATACGCTGGCATACTGAAACAAATCCTCCACGATTCGATATGGTGGGCAGATATGATACATATCCAGAAATTACTTTTACTATCAACAACTCTCTCTTGTCCTGGCATAACAATGGTTCTgtaagttattttttggtgagtacttatctaaacattttcctgatgtaacttaattttaataacacattttctcattccataaagGTTCCTTTCTCAAACTGTTCTGTTGAGTGCAAAGAAGGATTTTCGAGGGAACCTGATGGATTTCATAGCTGCTGTTTTCTGTGTAAAAAATGTCCACGTAACAGCTATGTGGATTATTCTCgtgagtactttaagtaatggatgacaaaaccttggcattccaccttcttcagtccactctaatttaataggggaaataaactgaaacttttgcaaatttatttgtcatagcaaaaaaaaaaaagaaaaagaaaaaaatagtacaaaaaaatctttgttagcccagatgatatctcattgaatcaaataacatgatttgaataattaaaatgatatcagagatgtattttgcacaatttctttattttatttttttaaaaggtaaatgtgtactttgacagataacagataacattgagtgattatgcttaaattattctgtagattataatgaataatccggtaactctttacaataaggttcattagttaaacattagttaatgtattaactaaccatgagcaatacatttgttactgtatttactaatcttcattaacgttaattaatgaaaatacagttgttcattgtttgttaatgttagttcacagtgatttaaactaatgttaacaagattttaataatttattagtaaatgttgaaattaacattaacaaagattaataaatgctgtataagtgcagttcattattagttcactaatgtagttaactaatgttaactgatgaaccttattgtagtgttacctaacaatcttttaaatttacatttaattaataacagctccagtataacaattatttatgaatataataattattataaaatttaatgaaatagtcaaaatttaactgttaatatttttaccttaaacattttaaattaattagtctacaaatatgaaataaattcacagataacaatgaaaaaaaaaaaaaaaaacttcaaataaatctcactaatgttaatgttcagatgaaatgtcaaaattaatagccactctaaaagttcaagaaaaaaaaaagatgaatacttgaataaagacgaatctttttcttttgtgccatctcctaatatatagttatgttatgttaagctctaatggggtttactggtgctccagtctccattccacaccataccacctgtaattatgtcattattttaacaaatcatgtccttattaactgcatggttaccaaactggaatttaaatctatgtgaatgaatttaccccaaatatttcccaccatcatgcatttataatcaagttctgtttgaccttgttcacgaaagtctgattttcatattatttttctgattctcagGGGATCCCTATGCCTGTTTTCCATGTGCAGAGAGTGAATGGTCTGATGAGGGCAGCGCGAAATGTAAGGCACGTTCTGTTGTTTATCTTCAGTTCACAGAAATCTCCTCCATCGCTGTGATGATTTCTGCCACATGCCTAATTATTCTCCTTTTTGCCATATTTGGCCTTTACACATATAACTACAACACACCAGTAGTTAGGTCTGCTGGTGGTAGCATGTGTTTCCTGATGTTAACATGTTTGATAATGTCTAGCATAAGTGTGTTCTTTTTCTTTGGACAACCCACATCTGTACATTGCCTCCTGAGAAATGTCATATTTGCATTTTTCTTCATTGTTTTTCTCTCCTGTATGGCTGTCCGTTCTTTTCAAATTGTGTGTATCTTCAAAATGGCTGCCAAGTTCCCCAGTGTTCACAGCCTTTGGGTAAAGTACAATGGACAGTGGCTCTTTGTTGGATTTTTTTCTGTCATTCATTTAATTTCTTGTGTGCTGTGGATGACTGTCTCGGCTCCCAAAGCTTTCAGGGACTCGTTAACTTTTAAAGACCAGATTATACTTAGCTGTGAAATGGGGAACACTGTAACTATTAGCATTGTTGTGTCCATGGGTTGGTTTCTTGGTTTCCTGTGTCTCTTATTTTCTTACATGGGAAGAGATCTGCCAAAAAATTACAATGAGGCCAAATCAATAACTTTCAGTCTTATTCTGCACAATGTGAGTTGGATTGTATACTTCACAGCATACCTCATTCTCAAAAGCCAATACATTCAGCTTCTTAATGCAATGACACAGCTAGCTAGTATATACGGAATTCTCTTCAGCTATTTCATACCAAAATCTTACATTATGATATTCCAACCACAAAAGAACACTCCTGCATACTTTCAAACATCTATTCAGAATTACACCCAGACCATTAGTAGGACTTAGaccacaacaaagcacacagtaaacacaaatgcttccttttaattagctttccttttaagaaattttctgtaaaatgcttttatactcatttccatactgtgtctttagatccactattaattcccaatcagatatcatttgtttgttgttatatctgcttgttgtggtgttgtatttgagttttgttaagaaaaagctatccccttttgttttaaaacactttaacaaggcagtgactggtgtgctggtagacattttaacttcatcatgccaacataagcctaaatggtaagacaggccgtcctaatcacactgcaatttaacaggtgaataaagaaattaaaaattaaaaattctgtcatcatttattcacaattatgttgttccaaacatgcatctctttcttctgtggaacacaacagaagatccttttaacattattgacagtgctttacaagaagactgctattctgttcacatagatacgacatctcaagagtctcaatatcatatttttaaccactgattgtagtagtacaaatatctatttgattcattattaaaaacaattcttccattataatgtgggatatatatgtaatatattctaccataataatcaaatgttgtaaattaataatacagtaatgagcccattttagttgcccatcagtgcttcatttggccacatgatgacatagctctatatatctttcaaggtcattatgttcagatgaaaatatgctaattgttgttttctgtcattccattgacagagacaacagaggtaggggagaccggcatggttgtaacaggtgtcagttgaaacacttccaatttcttcgttacaaatcacctggaatcacttcacatatgctcagtttagtccttattccatatgtgaagaagtagagccctacatatataggcatgttttctttgaatttcagtgtacctaggttttgaaagagaaaaacaaactgaggggtgtctctctgca

>gcT1R2C

tgaagacatgtattacaggtgccgttttatacttgcgggtactcagcttatgacatcatggactgtcgtccaccaataaattggcttgtttgtcatgctgatggtgttcccagtagcgtcaatatcagacactgtgtcaaagttccattgtattatttccaggagcagttgtggcctaatggttagagagtcggacttgtagcccgaaggacatgggttcgcgtttcagtaccggtaggaactgtaggtggggggagtgaatgaacagcactcccttccattctcattatccacaactgaggtgcccctgagcaaagcacctaacccccaatcgctccccgggcgctgcagacaaaaaacagctgagaggccttgagcaagacacctaacccccaattgctagtaaaccaattctgcctcaggtttcattgtactgtttcacaaacctagagggttgtctagtgacttatctcaaaataatataggaagcataggtgctgtgttatgaagtgacactggtggtttaatgtgacacttgtggggtgacaaatctagataaatgtcagatatacaaaaaaaacaactatatatatatttacaggaactgggttaagcaaatttaaaattttaccatatatgtgaacagtgatagtgttgctggccattcgaatcaatgaactttctattcactgcctctaaaccttagtgattctaatccaactgtaaacccctgctgagagttcagagagattgtttcacacaggtttgtaaaatttgttttttttttttatgatgagcattgtggtgtaatgctctatttaaaaactgactgacatatgccttgtgagtttagatatggaaatgggcgtttgtatcattgaacatggtttaagcaaattgaaatgagttttaagaaaattaagagtagaatataagcctgacactcaaacaactagccactgcacatccctgtttattgtccgacgggccatcttaaaccATGTGTCTCAGTAGCATTTACATTTTCCTTGTGGGCTTTACTAATTGTTTCTTTTTCAAAGTTTCTTGCTCAAAACATGAGTTCAGTTTGGAAGGAGATTACTTATTGGGTGGCCTTTTCCCTTTACATGAAATTGACCATGTGACACCCCTGTTCTCCCCAGAGACCACTGAATGTTTCAGgtaagcaaggatttttttatcagctgggttgtcagtatataatattttaaatcaaagtagcatttcctcaaagtcaaatttataaacaaaaactttaattacttcttcactgatgttttcaagGCACCATTTCTCAAAATCTGGCTATCAGATGTTGCAAGTAATGAGGTTTGCTGTTGAGGAGATTAACAACTCCACCACTCTTCTGCCCAGTGTTTCTCTGGGCTATGAAATTTTTGACCATTGTTCTGATGCAAAGAATTTCCCTTCAGTCTTAAGTTTTATCTCAAATAATGGATCAATAAAACCTAAAGAAAAACTCAACAACTATCAGCCTAAAGTGATTGCTTTAACAGGGCCATATGGAAGCACACCAACTATTACTATTGCACCACTGATCACAATGGACCTTATACCATTGgtaaattgtttctttttatacagtttacataacgaaatgcttacacacttactccctacactttgttcaggactaccaagttactatatatatatatatatatatatataatataaaaggaatgcacacacactccctccctaccgttcgggcaggacaagcaatttctctctctctctctctctctatatatatatatatatatatatatatataatgccaatgaattgtttatagtttagtgttttcttctttttcaaagGTGAATTATGGAGCTACTAGCTCTGCATTAAGTAATAAACTTAAGTATCCCTCTTTTGTAAGAACAATCCCTTGCAACAAAAACCTGATAGAGATGATTATTCACATCATACGGTGGTTTGGATGGAACTGGGTTGCCTTTCTTGGTAGCCAAGATGATTACAGTTCAGACGCACTAAAGCTGTTTAACGAGTATACAAGCAACACTGGCATTTGTTTGGCTTATCAAGAGAGTCTAAGCCTAAACGCAAACTACAGTCTAACACTTCAAAAGATTGATATGCTCAAGATCAATGTCATTGTTGTTTTTGCTGAGTCACAATATGCAAACAAAATTATCAAAGCAGCCATAGCAAACAACATCCAAGACAAAGTATGGATTGCAAGTCAAGCGTGGGCGATGAATCAACAGATTCCCAGACAGCCAGGAATTGGGAAAATTGGCACAATCATTGGTATTACAGAGAGATTGTTGTCAGTGCCTGGATTTAATGAATTTGTCTATAAAGACAGGAGAACAACTGATGTTAACCATAATGAAGAGGGTGACATCCAGAATATGAGTAAAACGTGTAATCAGGTTTGTGATTACTGCACATTGTTGACCGCAGAGGAGATTATAAATGAAAATCCCACATTCTCCTTTGCCATCTATGCTGCCATATATACCATAGCTCATGCTTTACATAAGGTTCTGCGGTGTGACATGAATGAATGCCGCAAAAACACAGCGGTTAAGCCATACATGgtgagtgaattcatttgaatccatgacaagttgaatcattgttttaattacattattgaataacaatgttttaattagttaaataatgaataatattggtcatatttttgagaaattgcatattacattataattattatacaataattggtaacattttattttgatggtcccttttgaacattctgttgattataagtaactttgcaactacatgtcaactaactctcaatagagaattagtagactatatgcttaatatctgctaacactttattttgatggtccaccaacagacattctactgactataagtaactttgcaagtgcatgtcaacttattctaacctctactaatatagtgttagtagacatgcaggtgcaacgttacttatagtcaacagaatgtgttaaagggaccatcaaaataaagtgaaacccaatcatttaataaataaatgaaacaaaagtaatattcattttatcaaagttataataataatattatgtggtctttttttattatggtaaaataaataaataaataaataaatcaccacaactcaacagCTTCTGGGAGAAATAAAGAAGTTGGATTTTCCACTCAATGGCCGTCAGATGAAATATGATGATAATTATGATTCAACCATCAGTTATGCAGTTGTGCTCTGGCACACTGATGTGAATCCTCCACAGTTTGAGATGGTGGGCACGTATGATACATATCCAGAAGTTACTTTTACCATCAACAACTCTCTCCTGCCCTGGCATAACAATGGTTCTgtaagtcttaatttgatgagcctttatctaaacatttaactttatagattccagtttttccaaagcaatcaattttaatcaattttgaaatactgacattaagactattagattgttctctgttgattgcgtatgcatgtatactactgaaaaagcacatttatcataaagctgacaaaagatctttttttctatgtttgaaagctagatatgagaaatttgtatgtgtttaagggaattatgtaatttgtattttgtaattcgcagtccgtttatggaaattgaatattgttcagatggttgagttgtttgagaatactgacctcagtttagagaaatgtgtaaaatcagttgaggaaaaactttaaatttaatgaaacattttcctattcttttaagGTTCCTTTCTCAAAATGCTCTGCTGAGTGTAAGGAAGGATTTTCAAGGCAACCTGAAGGTTTTCATAGTTGCTGTTTTACGTGTAAAGAATGCCCATACAATAGCTATGTGAATTATTCTCgtaagtacaaattaagtatccaaaaacattacatgcagtttttttgttgcaattttcacaagcaagtgctaaattttcaaaacatatcaaaaatatcattttcactacaaaaaataagtgtttaatctatataaatgttcattcacagtaactgcctttcatactatcaatcttttcataagcatctattcttgtttagtttaatacatttgtctatcatgtagcgtctgtgcaggatcagatgagcaagtcaactataaacttcgttaaattgttaccttcttgatcttttatttgtaaatctcacattggctccggcctggctgacattacaagctcaattgacatattttatggctgtgtcgacggatccacgaatctctcccttgtgacttcaaacgatcgcgataaactcgaattacggcaaaccatgcggccacattctagacgtcgtcaaactccaatatacgtacgcgcacacacagaggaacataccaggactctctttaagtcacattcattaaaatatgattaacatgtaatcctgtaagcctgtatttcagtttaatcatgtatttaattttaagttacgactatagttgtgtgaattgtaccccaaacctttatatgcgagtgtagaactgtatctttcccttaatgtctttgccacccagcatgtttgggtttgttttaaacctctctgaatgttctaattaacaatgtatgttatgttactgtaaaatgcatttttctattcttaatagtattgcgaagaaaacatactcagatctgacacttcataaactatgcaaattaggttgtaaatggagacaaagaaaaagtttgcccgccaaactttcaccctcgccattggctgttcacccaaggaggcggtttggtttgtttgtctttaaaagaaccacacacgtccttcctctgttgagccttttcatccactgagtcttctttatccgtcgcccttactcttcgttatctacgagacggactcttccttctctattttctggttattttactcttaagttaaaaccttgtttgaaagtcccgccgaagaaccctttctcggaaaaggaccaatcgctccagccgcacgaactgaaactcttgtagaaagacgaagcaaccacagcacctgaacctatgcaagtatagaaccattcttttcgaagcgttttaagcttcacgtttaagttgagtttccttgctggctctcaaaggttttaactgtttgtaattttgtaatttctttgatcacctctgttttcttgactttactttcgttttattctatgtatgttcgtttgttgtatttttagtcatatattcgtagttcgtatctattaaatacattatattcatgctctattgtttctgttgctcattcagaaaatcaagtcacttctacgttctgattttgcaaactgctttaaactttaacgctagaataggaaattattcttcatggccagaggaataactttcttttataatagtcaataaggaagctgatagtttgctggacgaactagaccagtctttctaaatttaactattaaactagaactaatcctgaaccatatatgtaattaattataatccgttgtaattaattcacaatatatgtgcataattccctttttggtcgatttatattagaattaatcataacgcgttatgatttaattatatatatatttcacccatgatttgagctaattgattaaatatctgtaattcgctacagaagattatttttggtggagaatgtgggcaacattttaaacttggttttgtgcgtgagcaactcagaataaatctgtgtgattattaatgttttatgcgcgctgttttcagtgagacgagacgcaacgtgctctgtaaacgtcaacaaactgctatttgtgtctaaacattttagcccatttgttaactgtttaaaataaactgcagttcattaatatttaaacttgttcctgcaaaagggagacaatctttttgcaacacagttttaatattaatttccgcttgaataagcaataacgttacctagtgcaaatctaatctgaaaaacagtttatctgatttatccgattgatgtgtaaaattggaagtttgttaacaccttgatccagtgttaatttgtaacacagcgaagaaatcctgcaaagttacatattcgtgttcactaaattgccacaaaaaagggatccgctgatcctaaaatttcatacccgtgataacttgcagttaaatcagccgtaagtgagtcaaatttccacagacaatttaaaagaatcacgagttaagaggaggttgatgcagtgtctctgaataaccaccagagagcagtctgtttaggtgtgtggcacaccctccctacctcattgttgtgtggactcattgacgccaccttgtgggcgttctgcgttaggtctgtttttcttacccttttctgtgtttggcattatttcaatttctcctgtggctattttaatgttgttttcatcccttttactttattgtttgctagtatttttaaatgtggtatactgatttaaatttggtttaaacacgttgaattgtaatttgaattaagtccccttttccaacaaagggaattcactttctggaagcgctcccccttgtggacactccccagataagtcaattcccttgccttttcgtctttcatttttacagttttattttgattctacttttttttttttttttaatttttaatttttctctttctttcttgaattattttcatttgggattccttccttttgatttcttatcgttgtatttcattcatgcttatttctttatcctgtttttttttttttttttcgttaatagccttttcacacctaagcctcccttattttatacacttattttaaatttaattaaacaaagtttaattgaaatttaaaattatgtttggtgtcattagttggcatttgctcatcaagaagcactcccctttgggggaattctcaaataaggaaggcctttttctcttttttcctcccattctgttgttctatgcatttatttaaattcaattgaacagagtttaattcaaatttgaatcaagtgcctctgaactgtttttactttgccttattgtttatttctgttctgcttttagctagttcattctgactgtcattatagaccccaattctcttgccctacacacttaattattcaaatttggtttaaacaagatttagactgagatttaaatttacaattaagttgtgtttatgtgaccattggtgtacttgcagatagtgccccctctagtgctcagtggtgagttgacggttgtttcagtgagttccagtgagtggagtaacctggcctggcggatccggcactggctgtgcatgaatcgtggacatttctgaccttgtgccaccggcaacccgcagagacatcagcaattgggtatctcaggtgcagggaggcaacgagtccaggctgacaacaaaggcaattttgtttaatttccctgttcaggcttctgcacgacagaaccgcccgtttggagtgactgcccattgaggggacagactctgggttcagggaaaactcaattttgacccgtcagtctggccatcggcacctcgcctgagtacatttgcaaatctaccaattgtgctaaaagtcactgtttgagtggttgcatcgtggtacaaaggggacagggtgcccaaggacaccgcagtcgagtgttggagcgtctaggaaaggttgcccccgccattgagtccttatctttgcaatcacaattcaccccaggcctagattaatgacatacagtccattcacttattgaaagccacagaatattagatattttctgatcatttaagtgttggccaaccccttttactcttaagccataccatccctctaggttttataacgcgacagcgccctcgtcagggcctggcataaacccttgccgtgataagaagcttaagtttcacttctcccctctagcccttcctattcttattttagttctattctattttattcctttttatttgtttattgttctgttttctcattaattgcctccattttgttctgttctatttgcttttctgcggtgtttgtttctttcagttcacgtaaaggcagagagccatcacggaagctgaataatagataaacaacctgagcagccagagtcattagaggaccaactaagctaccgcctgtcaaaatctttatttagatctggcactcacccacaaaaatcggcccacgtggtcattcccggctatcgacgataagtgtctggcctacattcaactaactgtcggcaccaattagttggaatcacacacaaagtttaccaaatcatgtattattgtaactcgttaatcccgcacttgcttgcattggttctgtttgtgctgtctttctctcgccaacgtgttcagatcgtcccagaagtttactacaaccaactgcgaagggcctacttcagagcacgtaacgagccaggaatggaggaagatttcaacttcataactctgtttcaaaatctgcaccctgcagtgaatcatcatcatctgggagttttggcctgcccacgcagtatgtctactcagcagctgcgagacttagcccacaaggcttattctaagcgaaaggctgcatctgaaaagactgttaaaaatccaaccatttatccggtctctgatcactgcccagaactggcccaagccattactgtggaaaatgatgtgagaattgaagccctggtagacaccagagcagatctcactctgatgtcatttgacctttttgaaagactcagagctgaagctaagagacaaaatagaactctcaaatcacaaagatgtgagctaaatgtgcagtcatacagccaaaatgaggtccaacttgaacaaatagcgcccatccacctcacaatcggttcaatgagcttggtacatcctgtgtacatatcacaaatggacacgtttccccttttcattggcaaagacctgctagcctgctttgagcccttattggactccagacagctgaaaatatgggctcaagtgcgagaacctctgcctttccagtcacccagaccacctgagccagactgtcaggtcacagaggtcacgggaaatctcggagctagccgcgggaaccccgtctcagccccagagcaaagttcaagttcgctactctgcactcttcaactcaccatagactctgactccttctgccccaaggtcatgacagtcacgacttccatgactttgaactcatgttaccagtcattggctcaataccatctgcaatgatcccaggagggagctcagacaacacagtgttcacagcacccttcaagatcatctccatcacttccatcatgctggtagccacagaaaaggtgtgcagaacagagctgatggaggaccaacacctcgctgtatacacagtctctaaccagcctgtctgagaaactcatgaaagtgctgcacctctgactgcccacatgacaattgacaccacaacggaggagccatttccaggatttgagactcaagtccaacaaactctgaaagaggcagatgctttaaagaacgacgcagaccaccaaaaactcagacaggtcctttacaagctcaaagcgtcagttgcaaaagactctttagactatggcctcactaacctccacacagtgcgtattccaacacacccaaatgctcctcccaccttcgtcaggcagtacaaaattcccatcgcctcacatgaaccagtgcaggagattattcgaaatacccaggatcaaaggagccactatcttctctacacttgatgtggcttcaggattctggaccatacccgtacatccagaagatcaacacaagcctttctggccttcacttttggcaaccgacagcacaccttcacatggtgcccattcggctatgccaacttaccagctgaattaaacatcttcctgaaaaaagcatgtccagttgcaaggatgagagggaatcttatttatgtagatgatgttctcattaaaaaaagcacaactgtggctgaccacctgaaagaaatagaccacgttctgaaccaattagccactgccggtgccaagactgccctccacaaaggacaatggtgccgaaccaaggtcagtgacgtgggtctacttatcggttcccagggcattgaaccgcagtccagccgcatccaagccattcaaaacatcaagcctcccactaatttctcagagctgcgcagtttcctaggtgtgtgcaactactcttgtcatagagaactactcggacattgcaagacctctaactgctctgctgaaaaaggactgcccatttgtctggacagaagcccaaaaaatttacgaactgaaacgacatttgtgtacagctccatttctggcttaccctgagccacagaaggagttctacctgcaagcaggattctccaaggactgcctgagtgctggcctttaccaacggtatgatcaagacaaaagagttgtagcatatgccagcaagacacttctaccagagtgtaaatactcaggctgtgaaaaggcattgctctgcactgtatgggccatccaaagattctccaactacatcggagcacaaaaggttatcctagagacctgtcaccagcctgtcacgttcctcaacagccaacgtatccgagatggcgtggtcaccaactcacgcatagccacgtggctgatggccctccaggggtgcaacgttgaggcacgatatgcccaaaaccacaagtctgcactgggcaacggcttggctactttccaaaactgctccgatgacacaccagcttcgacactggaggtagaagtaccccaacaacaacaacaaccaacctgtcacaagtacttcgaagagaatgcgtgccaaggtatgcccactgcctacgtcaatggatgttcctacaaccatgagggcatcctgaaggcaggtgcaggggtgttatggttaaacgaccaaccctgcccaccacaacatttcaagttgggtccccagtcgtcgcagtatgctgaagtagcagctattctcataacccttcgaatcgcctcggcccataacatcaaagaatgcctcatttgcattgactcaaactatgccagactcagtttcacatgccaccttctggtctggaaacaaaatggcttcaagaccgcaaacaacaacacctgttccaagcatgtgatgacatcacccaaacacacaacatgatcgtttactggaaaaaggtgaaaggacactcatagcaaccagggcatgataaagactcgaatgaccaaactgatgcccttgccaagactggcgcagtacatggcaatctctcgtcacccccaccccagtcgcccacccttagtgtggcagctaaaacccgcagccagcgaactgttcctacaacagttcccacctcacagccactgtcaatggctccacaaattccaacaatgatatagctgacatccgagcctccgacacctccataaaagctgtattaaaccacctctcagacccctccaaccacccgatcacggcaaattattcttcatggccagaggaataacttccttttataatagtcaataaggaagctgatagtttgctggacgaactagaccagtctttctaaatttaactattaaactagaactaatcctgaaccatatatgtaattaattataatccgttgtaattaatccacaatatatgtgcataattccctttttggtcgatttatattagaattaatcataactcgttatgatttaattatatatatatatttcacccatgatttgagctaattgattaaatacctgtaattcgctacaatcatttaatccaatagatgactactaagtgatgtgtttttttgttgttgttttttttttgaaaatctaaaatatcagaaagttttcagtgatgggtaggtttaggggtagggttagtgtaggggggatagaatatacagtttgtacagtagaaaaattatgtctatggaatgtccccaaaaaacatgaaaacccaacacagtttgttttttcagtatagactgacaaattactttaacccttaatctgaaaggtcaactacaatatagattcagccaaaagatacagtgcagaatatgtgggtggagtaattattgaaagtttagatagaggaggggaaattgttgctgctccactccactcactctgatacagtgagaacatttgcaaactctgttgtagctcaaaacctcaaggccgtatcatatcttcttcacatcttttaatctcacactggatacatactatcacaaacattatgtattttactttaaaggcacaatgtgtaagatttttggattaaaatatccaaaaaccactagaacaatgttatatattttgttgatgtttgtacttatcccaaatgtttccaagaatgtttaaatccagaggaataagcaattttaaccaggacacagaccgcgtccgtgcgtcgcgcatcaatgaccgttacccttgatttcctcaactgtttggatggatacattcatcgacagaaaaataatcatgttatatagctcagcacagtaagtcttattgtttaaattgcgtttgcttgatttacagcgagtaccatgttttaccatacctaatatcgatctagcttactgcattgtgtgttaagtgtctcatagtagctgccgagcgaacgcagagaaactattacaactttccacacacaaatgtatctaatatgataaaacagcgctgcgttatcccacatatgcttgaccggaagaagcgggagcggtgactgcggcataataaaagttcagctgctctcgaggcatgtgtcacgctcgtatctcattagcactcaatccagcggcctcgttcagctcccacagcactcggccctgctctgcttcatactacagtaacattaataatctcaatgagtcccgtcctccacgaaatcaaggcgtcatcaagctatgcctttgttttgaataagcgacctctagcggtgaaaatttacatattttgcctttaagtcagttacataagtaatgcaacaaggctgactattgaaactaattattatgtggaaagaaaacatgtatagattttgaaagaatgactagctgtgttacaagtgtgaacagtttggatttttgtaggttagaatatccaccttatgaaaatactataaaagtgaagttttttaaataatataattattagtgtaatgattactctttttaaagtatgctgaagtgtactgtacttctttttcacaaagcttttagaaaatactattataaacattcatattattttttcagGGGACCCCTATACCTGTTTTCCATGTGCAGAGAATGAATGGTCTGATGAAGGCAGCACAACATGTAAGAGACGTTCTGTTGTCTATCTTCAGTTCACAGAAATCCCCTCCATCGCTGTCTTGGTTTCTGCCATATGTATAATTATTTTACTCATTGCCATATTTTGTATTTTCGCCTATAATTACAACACACCAGTGGTGAGGTCTGCTGGTGGTAGCATGTGTTTCCTGATGTTGACGTGCTTGATTTTGTCTAGTATAAGTGCATTCTTTTTCTTTGGAGAACCCACATCTGCATTTTGCCTCATGAGAAAGGCCATATTTACATTTTTCTTCACTGTCTGTATTTCCTGTTTGACTGTCCGTTCCTTTCAAATTGTTTGTGTTTTTAAAATGGCTGCTCAGTTCCCTATGGTGCACAGTCTTTGGGTAAAGCACAATGGCCAGTGGCTCTTCATTGCATTGGTGTCTGTCATTCATTTAATTTCTTGTGTTGTATGGATGACTTTCTCTCCTGACAAACCCATCGCCGACTCATGGACTTTTAAAGATCAAATTATGCTTATCTGTGAAATGGGGCATGCATTAACCATAACCATAGTTGTGTTCATAGGTTGGTTTCTTGGTTTCCTGTGTCTCCTGTTTTCCTATATGGGAAGAGATCTGCCAAAAAATTACAATGAGGCCAAATCAATAACCTTCAGTCTAATTTTGTACTATCTGACCTGGATTGTATATTACACAGCATACCTCTCTTTCAAAAGCAAATACATCACTCTTTTGAATGCAATTGCCCAGATATCCAGTATAAATGGAATTCTCTTTAGCTATTTCATACCAAAATCTTACGTCATAATATTCCAACCACAAAAGAACACTCCTGCATACTTTCAAACATCTATTCAGAATTACACCCAAACTATCAGTAGGACTTAGacattagtcgttgtatagtcaccttattttaaacgtaagagtagtgctgccatttgtaacttgaatgtgcaaggcttccggtctcatctgcttccagttccctttcaaatgggaactcgcactgtgtcaaagacactttgggggaatgccttggcgtgacgatgtctgaagtaagaatagaaacgtgacaatgacattggcctgtgacagcccgtgacgtcactctagtgtgaccgggagtataaaaagagcacctgaagtacacgtcattagcttttttgtcttcaggtgaccctttgtttgaagtatgtttgggcgactattgccatggcatcctcctctgacaagcactatctgacaccggacgatacacatcatatgtgtgttatttgtcttggtgaggatcatgcgcgttccgttctcgagggagcggaatgcgcagattgtgagggcttttccatgaaaaagctccattcacacctggcacttttctcgagggaatcggaacaggcatcagtccccaggggatcaggacctgctgcagctgaggcacagtggagattggtcgagctggcggagaactttgaacgaggtatgtctctttctcattgctctgctgccgacgtgagcgctgatccagatgaggacgttctctcattagaatcatccgatctggtggatagtgctcttctggcttcaagccatgagaggttgagaggtcttggaagaaaccatattcagcttgcatttatccctttcagcattcaaattatgtgaatgtggagggattgcgagagcatggatatgcatcgatgcatcccattgaagagacgttcgtgggctatcttgcaatgagtaatgcatcgatgttaaagactccaaccctgccatccaagccattaaaaatgacatctaagctgaatggcagggcgtatgcggctgcaggtcaggccggtggagctttacacactatggccgtgttacaagcttaccaggctga

>gcT1R2D

ggttgtgtaataaatgccaagtgttctagtaaaccagttgtgcatcaggattaattgtactgttccacacacctagtgtcacaagtatgctctgttttgtttattttctgtgttcctgttggccttagttccctagttagtaaccttggttcttagttagttaacacctgttctgttttagcttgcatattctccttgtgtatttaagcacttagtttcctcagtttccttttccggtattgtttgttaatgtgtagttatgttgatactcctgcggtttctcctttcttaaaagactcttggtttacttttgaatcatgcgtgttcatcgacacattatacctagtgtagaactgctacacagttctcatatgatttcttagaagtttgtgttatgaagtgacactggtggtttaatgtgacacctgtggggtgacatatagaataatgtcagatatgcaaaaacatttaaatgtatgcaggaactgggttaagcaaatttaaagatgtaaagtatatgtgaacagcaacagtgttcctagccattcaaaatctatgatttcagcattggttttcagcctccatggagagcattaactatattatatacttaaatgtgatgtgtaacaccacagtactcaactcaacagaagctgcataaaatctcatgtatagacatgccagagtccattttaatttttgctcaccacctttaaactttgaaaccttagtgattctaatccaacagtaaagccctgctgagagtttagaaggttgcagtttgtaaaatatgttagaaatgtggtgtaatgctctatttaataactgattgacatatgctttgtgagtttagatatggaaatgggcatttgtaccattgaatatagtggtttaagcaaattgaaatgtgttttaagaaaattaagtctatatgagcctgactcacaaatgactagccactgcacatccctgtttcatgaccaacaggccatcttaaacaATGTGTCTCAGTAGCATTTACATTTTCCTTGTGTGCTTTACTATTTTTTTCAAAGCTTCTTGCTCAAAACATGAGTTCAGTTTGGAAGGAGATTACTTATTGGGTGGCCTTTTCGCTTTACATGAAATTGAACAAGCAACACCCCTGTTCTCCCCAGAGACCACTGAATGTTTCAGgtaaggaaggacctttttatcagtcaagttatcaaaatgcaatattgtaaatcaaagcagcatttccttaaagtcaattttataaagtgaaaattaaattacatgctattaacttcctcactgatgttttcaagGCACCGTTCCTCAAAATCTGGCTATCAGATGATGCAAGTAATGAGGTTTGCTGTTGAGGAGATTAACAACTCCACCACTCTTCTGCCCAATGTTTCTCTGGGTTATGAAATTTTTGACCATTGTTCTGATGCAAATAATTTCCCTTCAGTCTTAAGTTTTATCTCAAATAATGGATCAATAAAACCTAAAGAAAAACTCAACAACTATCAGCCTAAAGTGATTGCTTTAACAGGGCCATATGGAAGCACACCAACTATTACTATTGCACCACTGATCACAATGGACCTTATACCATTGgtaaattatggttctttttatgcagttttgtactaccctttgttcaggactaccaagttactatatatgcagtataatgctaatacattgtttataatattttgttttcttctgtttcaaagGTGAATTATGGAGCTTCTAGCTATGTGTTAAGCAACAAACTAAAGTATCCCTCTTTTGTAAGAACAATTCCTTGCAACAAAAACCTAATAGAGATGATTATTCACATCATACAGTGGTTTGGATGGAACTGGGTTGCCTTTCTTGGTAGCCAAGATGATTACAGTTCAGACGGACTAAAGCTGTTTAACGAGTATATAAGCAATACTGGCATTTGTTTGGCTTATCAAGAGAGTCTAAGCCTAAACGCAAACTACAGTCTAACACTTCAAAAGATTGATATGCTCAAGATCAATGTCATTGTTGTTTTTGCTCTGCCACAATATGCAAGCAAAATTATCAAAGCAGCCATAGCAAACAACATCCAAGACAAAGTATGGATTGCAAGTCAGTCATGGGCGATGAATCAACAGCTTCCCAGAGAGCCAGGAATTGGGAAAATTGGCACAGTCATTGGTATTACAGAGAGATTGTTGTCAGAGCCTGGATTTAATGAATTCGTCTATAAAGACAGGAGAACAACTGATGTTAACCATAATGAAGAGGGTGACATCCAGAGTACGAGTAAGATGTGTAATCAAGTTTGTGATTACTGCACATTGTTGACCCCAGAGGAGATTATAAATGAAAATCCCACATTATCCTTTGCCATCTATTCTGCCATATATACCATAGCTCATGCTTTACATAAGGTTCTGCGGTGTGACATGAATGAATGCCGCAAAAATACAGTGGTTAAGCCATACATGgtgagtgaattcatttgaatccatgacaagttgaatcaatattttaattacattattaaataacaatgttttaattaattatataatgaataatattgttaatattttttatgaaattgcatattatattattggattattatataattaataaatgaataaaacagaataaaacaaaggtaatattcattttactaaagttatagtaatattaaattggctttttttttttttttttttttcacaacagCTTCTGGGAGAAATAAAGAAGTTGGATTTTCCACTCAATGGCCGTCAGGTGAAATATGATGATAATTATGATTCAACCATCAGTTATGCAGTTGTGCTCTGGCACACTGATGTAAACCCTCCACAGTTTGAGATGGTGGGCACGTATGATACATATCCAGAAGTGACTTTTACCATCAACAACTCTCTCCTGCCCTGGCATAACAATGGTTCTgtaagtcttaatttgatctaaacatttaactttatagattccagtttttctcaattaactcaaccatcaaaacagactcctacttcagaaaaacaatccagtttatcttcatgatgtagtactgcattttttttcagctacagtagcataaaatacagcaaatactgacatcaattagattgttctaaattaattacatatgcaagtatagaaaaagctcatttatcataaagctgacaaaatatcttttttctgttttaaagcaagatctaagaaatgtgtgtttgtttaagggaattatgtaattagcattttgtaattcgcagtctgtttatggaaattacgtagtgttcactgttcagatggtcgagttaattgtttgtgtaaaatcaaataagaaaaactgactttattaacacatttttcctattccttaaagGTTCCTTTCTCAAACTGCTCTGCTGAGTGTAAGGAGGGATTTTCAAGGCAACCTGAAAGTTTTCATAGTTGCTGTTTTACATGTAAAAAATGCCCGCGTAACAGCTATGTGGATCATTCTCgtaagtacggaagtggtcattcacaccttgatatttaagtttcttctgtccattctctcaccacaatacactttacttaagatatgttttttttttaatgtaatgtgaagacaaattttcccacatccatttaatttctagttctgtcataaaactaggtcctagaaaataggtccttaatgtaatctgctagctagcaccattcactacatggtacaagctgcagccattgagcttggtgcttaacataaatagtttggttctgtgtttgctgaaagcaagtcttgagaaacagaaaccctccaccttccccagctcagctgaaataaaatatttttttggtacaatgtagagtatgtgagcagagtggagtaattattgaggagggaaacttgttgccactccactctggtacagtgagaacattcactgtattggcagtaaactctgttctagcacaaaacttctaccatatcttcttcacaccttttcataataacacactggatattacaaatattatggattttactgtaagccagttacataagttaggtaatataataaagcaataagccccaaaaaagccgtggtttacagtgaatttataacagctaagagtggagtgcctaaaacccccttagctgttataaattcactgtaaaccacggcttcacggggcttattgcttttataaaacggttcccacacaatacaaatattaaagccaaaaaaatatgtatcaatgcaactttcatgaagtaaaatcattaaaaggcttccttccgctggaaaaaatatttcctgaccgtgaacagcaacagaagttacatttattacgccattagatggcgacaaagattgtctttatgagtgagtcacaaaaccttttatattaaaacttgttgtgaacacggatcaagacgcaaatgacaaatgctttgactagcgctgtcagtgtcagcagggcacgggaaaacccattaaatgttaaaaggacaagattgcagcggacattcaacaaattttttattatgaacataggacttgacctgaaggaaaaatgctaaatttgaatgcaggtgatacactcggtcactctgtctctctcacaatactcttctacataatgcagtaagcttcaatgaacaaaatcaatagagaacaaacatgtttacgttgttaagagtggttgctaagacagacgcagcaatacttatgtaatgcagtcagctgtatgtttttgggaattttacaactgcttcgaatgtggctcaaccaatcagaatcaagggcaggaactatccattttataagtgtattctttaaatacaattgtagcgtgtattactgtaacatcttcagcaaccaggttaatttgaaatgtgccttgtcacgcaggggttgcaaacaaatggaaataaaagagctgaatctatttgaaaacattttatttattaacatttattaacatttattcctttttaatcaaaacccaaaacccaatttcaggttatcttgaaacaaaaacatgagaaacagaacacctaatacaatgatgacatcagaccaagactaactgaaatattgaaatactcaagactaactgaaatactgtgtgtgtgtgtgtgtgtgtgtgtgtttctgtaaaatgcgaaaaaaatcaatttcataaagacatcattggagtttttaatatttttatattgtcgtaatttcacagttgtttctacattaatttggaaaaataacttaaagacagtatatttaaaaaaaaaaatttaatagcatctttttatgaatgaaggccagtatgtctgatactaatactgtcattggggccgaactcaagacaggaaggttccacagttagagcctgtagatctcccaactcgcttgactgatgctagagccaacaagagggcagtcttaagtgagagggaccgaaggtcggccatcttgagaggctcaaagggagggcctcggagtgccctaagaactgtggagcggtcccatgaagggactgagcaacggcaggaaggattcatccttctggcccctctgagaaacttaacgaccaggttgtgcaggtcccacaactccagttgagggtgccaaattgtgaaggtctctcctcagcggtataggccataggtgctgacagcacctggactaattctgggaaccaaggcagattctgccagcatggtgccaccagacgaactgaccatttctcctctctgatctgaccgatgagttgtgggagcaaggctaccggagggaaagcataagggccttttaaacaaagctgtagtggtcgcatgtggagaaggcccagaagaactaaggaagccgctgccatgaggcccggcagcttccgaaacctcttgaggggaagaagggatccttccctgaacgaggccgccatgctcctgattttcaaggcaccctcttgtgagagccaagcctgcacttgagttgagtcgacgacagctcccaggaacaaaattcgtcggctggggaacagtgagattttgtcaaagtccagatggttaaggagcagggatctgtgggcgcatagctcctgtctcaactcggccatttgtcgagatagttgagtatgtggatttccatctgtcagaggggaaagagccagggcgaagctgcgcttcatagttaacattctgaacggccgtctcatgagagcacgattcagatgtctcagatcgagaataggcctgcggccgccatctttcttggggacaaagacatagcagctgtaaacacctgacttgctctcttcacagcacctttttccagcagagcctgaactttggctctaaggacatgagaactgctgtctaagaccgaagtgtgaatttttttgtacatgtaagtgttttgaaaattttcaccttgtgaaaatagtactaaagtaaataaaaaactaagtatattcttttaaaggatattattactgtaataattattcgtttgaaaagtatgctgacgtatactgtatttctttttcacaagcgtatgggttttggcaaaacactattataaacattcatattattctactgtttttcagGGGACCCCTATACCTGTTTTCCATGTGCAGAGAGTGAATGGTATGATGAAAACACCATGACATGTAAGAGACGTTCTGTTGTCTATCTTCAGTTCACAGAAATCCCCTCCATCGCTGTCTTGGTTTCCGCCATATGTATAATTATTTTACTCATTGCCGTATTTTGTATTTTCGCCTACAATTACAACACACCAGTGGTGAGGTCAGCTGGTGGTAGCATGTGTTTCCTGATGTTGATTAGTTTGATTTTGTCTAGTATAAGTGCATTCTTTTTCTTTGGAGAACCCACATCTGCACTTTGCCTCCTGCGAAATGCCATATTTGCATTTTTCTTCACTGTCTGTATTTCCTGTTTGACTGTCCGTTCCTTTCAAATTGTTTGTGTTTTTAAAATGGCTGCTCAGTTCCCTAAGGTGCACAGCCTTTGGGTAAAGCACAATGGCCAGTGGCTCTTCATTGCATTTACTTCTGTCATTCATTTAATTTCTTGTGTGATATGGATGACTGTCTCTCCTGTCAAAGTCACGGCTGACTGGTGGACTTATACAGATCAAATTATGCTCGTCTGTGAAAGGGGGAATACTATAACCTTAACCATAGTTGTGTTCATAGGTTGGTTTCTTGGTTTCCTGTGTCTCCTGTTTTCCTACATGGGAAGAGATCTGCCGAAAAATTACAATGAGGCCAAATCAATAACCTTCAGTCTAACTTTGTACTATCTGACCTGGATTGGATATTTCACAGCATACCTCTCTTTCAAAAGCAAATACATCATCCTTTTGAATGCACTGGCTCAAATATCCAGTATAAATGGAATTCTCTTTAGCTATTTCATACCAAAATCTTACGTCATAATATTCCAACCACAAAAAAACACTCCAGCATACTTTCAAACATCTATTCAGAATTACACCCAAACTATCAGTAGGACTTAGacattagtctttgtgtatccaccttatttttaaagtgctgccatttgtaacttgaatgtgcgaggcttccgtctcatccacttccagttattttacatgtacaaaaacatgtatttgttatcatattatttgaatcttttatcataatcataaacactgatttgtagcgcaaagttttactgtttaatgcactttgtttcttctaattatttacttatagtagctgaataatcggatttggaagtctcacacattcacggaaattaacttactttctagttgcaaaataaaggataggatgtgaactcaaactctgttagtggcttagatccttccctgaattttaagaatgaaaagagtaatttaaataattataaaacttttgtttacatgctagcattgacatttaagtgtaatgtaatgtaaacacaaatgcttgcttttaacttgtaataaagaacactttcaaaactcccataacttagtgtccaggtaatacagagatttgtgatgtttggatgtttgacatcatactgactgcctattaatgtatttggcatgtcccagttttgctttaaagagcatgttttatgcccctttttacaagatgtaaagtaagtaacaagatgtaaaatgtccccggagtgtgcatgtgaagttttagatcgattttttttttttttatttatttttttatagcatgttaaaatggcaacttttggggggtgagcaaaaatatctaggaaatgtctagaaacgtactgaaggagggaatggagatgttactttgatagtggccagtgagattatacaggtataaatatgaccggcattattcacttcattcatgttttgctggggaggcgagacaagatcccggccattcagtggtggttcagggctctggcagagggacgtaacgtctctgttccatccttcagggaacggggttacattagtaacctggacattcccattcagtagggctgcaacaactaat

>gcT1R2E

tagacatttgaagaaaataaaaatatttatcttttattttattgtatccattacagtcaaactgtgtgtgtatttggtgttgatctaatcttaatttggggtatacacgccgggttatatatacagggttgtgtaataaattgttctgatttttacaaaggaatgaatgtataatgagtttatttaagctggacaatgacaccattttctttaagagctgctgtgcagccaaaattatttgccagttatcactgtaaagctgctttgacacaatctgcattgtaaaaagcgctatataaataaaggtgacatgacttgacttaataaatgccaagtgttctagtaaaccaattgtgcctcaggtttaatactgttccacaaacctagagggatgtctagaactgctacacagttgtcatatgatttctgtgacacttgtggggtgacaaatctacagtaatgacaggcatgcaaaaacatttaaatgtatgcaggaaatgggttaagcaaatttaaaaatgtaaagtatatgtgaacagcaatagtgttgctggccattcataatctataaattagcattggttttcagcctccatggagagcattaactatattttatactcaaatgtgatgtgtaacaccacagtactcaactcaacaaaagctgctgtatacacatcccagagtcattttttttaatattttctcaccacctctaaactttgaaaccttagtgattgtaatccaactgtaaacccctgctgagagtttagaaggtttgtaaaatattttataaatgtggtgtaatgctctatttaataattgattgacatacgctttgtgagtttagatatggaaatgggcatttgtatcattgaatatagtggtttacgcaaattgaaatgtgttttaagaaaactaagtctagaatataagcctgactctcaaacgactagccactgcacatgcctgtttattgaccgacgggccatcttaaaccATGCTTCTCAGCATTTACATTTTCCTTGTGGGCTTTACTAATTGTTTCTTTTTCAAAGTTTCTTTCTCAAAATCTGAGTTCAGCTTGGAAGGAGATTACTTATTGGGTGGCCTTTTCCCTTTACATGAAATTGAACAAGCAACACCCCTGTTCTTCCCAGAGACCACTGAATGTTTCAGgtaagcaaggattttttatcagccgggttatcaatatgcaatattgtaaatcaaagcagcatttcctcaaagtcaaatttataaaatgaaacttcaattacatgctattaacttcctcactgatgttttcaagGCACCGTTCCTCAAAATCTGGCTATCAGATGTTGCAAGTAATGAGGTTTGCTGTTGAGGAGATTAACAACTCCACCACTTTTCTGCCCAGTGTTTCTTTGGGCTATGAAATTTTTGACCATTGTTCTGATACAAATAATTTCCCTTCAGTCTTAAGTTTTATCTCAAATAATGGGTCAATAAAACCTAAAGAAAAACTGAGCAACTATCGGCCTAAAATGATTGCTTTAACAGGGCCATATGGAAGCACAAGAACTATTACTATTGCACCACTGATCACAACGGACCTTATACCATTGgtaatttttttctttttatacagtttacataacaaaatgcttacatacttactccctaccctttgtttaggactaccaagttactatatatatatatatataatgcttttgaattgtttatagtttagtgttttcttatctttcaaagGTGAATTATGGAGCTTCTAGCTATGCGTTAAGCAACAAACTAAAGTATCCCTCTTTTGTAAGAACAACCATAAGCAACAAAAACCTAATAGAGATGATTATTCACATCATACGGTGGTTTGGATGGAACTGGGTTGCCTTTCTTGGTAGCCAAGATGATTACAGTTCAGACGGACTAAAGCTGTTTAACAAGTATATAAGCAATACTGGCATTTGTCTGGCCTATCAAGAGCGTCTAAGTCTAAACACAAACTACAGTCTAGCACTTCAAAAGATTGATATGCTCAAGATCAATGTCATTGTTGTTTTTGCTCTGTCACAATATGCAAGAAAAATTATCAAAGCAGCCATAGCAAACAACATCCAAGACAAAGTATGGATTGCAAGTCAGTCATGGGCGATGGATCAACACATTCCCAGAGAGCCAGGAATTGGAAAAATTGGCACAGTCATTGGTATGACCGAAAGATTGTTGTCATTGCCTGGATTTAATGAATTCGTCTATAAAGACAGGAGAACAACTGATGTTAACCATAATGAAGAGAGTGACATCCAGAGTACAAGTAAGACGTGTAATCAAGTTTGTGATTACTGCACATTGTTGACCCCAGAGGAGATTATAAATGAAAATCCCACATTCTCCTTTGCCATCTATGCTGCCATATATACCATAGCTCATGCTTTACATAAGGTTATGCAGTGTGACATGAATGAATGCCGCAAAAACACAGCGGTTAAGCCATACATGgtgagtgaattcatttgaatccatgacaagttaaatcattatttaaattacattattgaataacaatgttttaattaagtaatgaataatgttggtcatatttttaagaaactgcatattataatatattatttaattagtatacaataatttcataaatgaatattattataagtggtctttttctttttacagCTTCTGGGAGAAATAAAGAAGTTGGATTTTCCACTCAATGGCCGTCAGATGAAATATGATGATAATTATGATTCAACCATCAGTTATGCAGTTGTGCTCTGGCACACTGATGTAAACCCTCCACAGTTTGAGATGGTGGGCACGTATGATACATATCCAGAAGTGACTTTTACCATCAACAACTCTCTCCTGCCCTGGCATAACAATGGTTCTgtaagtcctaatttgatctaaacatttgactttatagattccagtttttctcaatcaatttagcaaatttctccaaatgaaagccaatgctctcaaaacaattaacacaaccatcaaaacagactctttcttcagtaacataaaatacagcaaatactgacatcaagactattagattgttttctattgattgcatatacaagtatacaattgaaaagctaatttatcataaatctgacaaaagatttgtccatgaaagcaagatctgagaaatatgtgtgtgtgttaaaggaaattatgtaagtagtattctgtaattcgcagtccgtttatggaaagtatgtactgttcagatggctgagttaattgtttgtataaaatcaaataataaaaattgtaaatttaataatacattttctcatcccttaaagGTTCCTTTCTCAAACTGCTCTGCTGAGTGTAAGGAGGGATTTTCAAGGCAACCTGAAGGTTTTCATAGTTGCTGTTTTACATGTAAAAAATGCCCGCGTAACAGCTATGTGGATCATTCTCgtaagtaccttaggaagtggtcaatgacaccttgacatttaagtttcttctgttcttccattctatcaccacagtacactttactggagatatgatgcttttttaagtgtgatttgaagacaaaatattctcccacattcatttaatttctcattctgttgtaatactaggacctagaaaaaaggtccttaatgtaatctgctagctagcaccatttactacatggtacaagctggttgcatctgcagtcagtgagcttggtgctcaacatttaattagtttggttctgtttgctgaaagcaagtcttacagcaagaaatcagaaaccctccacctttcccagctcagctgaaatgaagtttttgtttgtttgtttgtttgtttgtttgtttttttgtacagtgcagagtatgtgagcagagtggagtaattattgaaagtttagatagaagagggaaattttttgccgctccacaaccactacacagtgagaacaatcactgtattggcagtaaactctgttctagcacaaaacctcatggccataccatatcttcttcacaaccttttcataacacactggatggaacattacaaacattatggattttactgtaagtcagttacataagttaggtaatgtaagtgtttaatacaactgtagtgtgtattactgtaacatcttcagcaaccaggttcatttgaaatgtgtcaaaaaccacgttttcaaaatcaaaatttaaaaaaatcaaaaaccaaattttcacttcaggttatcttaaaacaaaaacacgacaaacagaatacctaatataacaacaagatcaagcaaagactaaaataaggactatatatacacgcagagaaataccagggacaaaacaaaatagttaactcgatgcaggtgagacacatgagggcaaatcaaacactatggcaacagatgacagatataaagaaaactgaactgggaacagtgcaaaactaacaggctaacaacttaaaacaagattcaaacccagaaacatacgtaacattaacacccaatcaaaatggtgcatccacatgccttaaaacatcaacaagagtacaacataggagggtggacaggggcttaggaggaggatgcagaactggcagacgggacacaggacgagggaggagccagggaggagacgatggctgggggagccagggagtgaccccaaacagttctagcaggctgaagacccacggtggagccgatggagggaggagccatggtggttccttggtgaagaggcagagcaaaaccagggggatgacttatagaggtggggccagtggttgtggagacccaggctgcgccaaagagccgaagagaccggctgacaacaaatgacctggagacaaaggcagagccgcagtgacaagcggctgaggaggagccagggtgacagaggactgaggcagagccggtgtgactgagggccgaggtggagccagaggaaaggaggagccagacagagccaaagggtggagggacgaggtgcagacaaaggtccagaagtccgtggcaaacgcagagcgatgactgaccaaggcagagccggaggtacgaggaagcccatttgagatagaaggacgacggtcccagttggagctgtgggagattggttgatgggctgaggtggggtgacgggcttggaggtttgaggtagagctagagagtcaacacaccagggcggagctggtgaacaggaagcccaaggcagatccaagcttctaggagccagcgaaggagtcaaaggactgaagggagctagcggagacaggaccaaagaactggattgcacgggcagagggttacgagatggcctacttggccatgacagggcaggcagtgagttcaaaaaataaatacaggataggcagaggaggtgggagtgggaggcagggtgggaacatcaaatcacactgatatgatgctaggcaatgaaacatacttggtacttggcaacaaaacagacttggtgctgggctacgaaacagggatggtgcagagacttacatactcattggggttcaaagtcagcacactcatatcagccttggtactctgggtgaggctccattccatgccctcgtactctgacccacaggaacagacacagggactggctcgcacagctggtcagactccttttctggctcaggctctgggacaaacttggcttcgggtcatgaggtcttctaatgccgtggctaatggagcaatgtatgctgcagtcgctagcggattttgatcctcctcactctcaccgttggagatgagggatccgcactgatgtaaaatacactcaaagtacttgtggaatttccctcaagggccgcctggttccctttgtgcaatatgatgacatcagcctctgcgtatttttatttttaaaatacgcagagtttgatgtcatcatagtgcatgaaaggaaccagatctcgaaacctctttgtatgttactcaagggacgcatccccctgggagaggaagaggatctgaactgctgttagatccttttgatcggtctgatcttctgttacgttggagtttcaaataaacggaaataaaagagttggatgtattttcaagcattaatcctttttaatcaaaataaaaataatttaaactaaaaactaactgaaaataaggactatatatgtacacagagaaacactagggacaaaacgagataattaactagacacaggtgagacacatgagggcaaatcaaacactatggcaacagatgacagagacaaagaaaactgaactgggaacgatgcaaaattaacaggattacaactcaaacaaaactcaaacccagaaacagacataacacttgcattatttgctactgaattaactgattgttaagtactaatttgaaagagctgttgtgttttagtgattaaaccaaatgttctcattacatattacaatgatcttgtgttttgaaacaattatgtgaaaagaaaacatgtttagctgttttaaaagaatgaatagcagtatgaacatttttgtaccttgtgaaaatagtactaaagcgaataaaaaagtgtaagtatattattttaaaggttattattacgtggcgacagccacgacacagttcctcaacagccgtgatgaatacaatcctcaattggatggaactggaataaatactttgaatgttgcgatcctgtctgacttatgatagctacctgaatcgtaacaaagcactgttggccagaggagaactggccccccgactaagcctggtttctcccaaggtttttttctccatttaaacacctatttgccacttgtctgccacctgatgtcacctgatggagtttgggttccttgccgctgtcgcctttggcttgcttagttggggacacttgacatttgacttgacatttgatattcaacagtgctttgatctgcctgcattgacactattcttttaagagctgctgtgcagccaaataatgtaccagttatcaatgtaaagctgctttgacacaatctacattgtaaaaagcgctatataaataaaggtgacttgacttgacttgactgtaataattattctttttaaaagtatgctgaagtatactgtatttccttttcacaagcgtatgggttttggcaaaatattattataaacattcatattattctactgtttttcagGGGACCCCTATACCTGTTTTCCATGTGCAGAGAATGAATGGTCTGATGAAGGCAGCACAACTTGTATGACACGTTCTGTTGTCTATTTAGAGTTCACAGAAATCCCCTCCATCTTTGTCTTAGTTTCTGCCATATGTATAATTATTTTACTCATTGCCATATTTTGTATTTTCGCCTACAATTACAACACACCAGTGGTGAGGTCTGCTGGTGGTAGCATGTGTTTCCTGATGTTGACGTGTTTGATTTTGTCTAGTATAAGTGCATTGTTTTTCTTTGGAGAACCCACATCTGCACTTTGCCTCATGAGAAATGCCATATTTACATTTTTCTTCACTGTCTGTATTTCCTGTTTGACTGTCCGTTCCTTTCAAATTATTTGTGTTTTTAAAATGGCTGCTCAGTTCCCTAAGGTGCACAGCCTTTGGGTAAAGCACAATGGACAGTGGCTATTCATTGCATCTGTGTCTGTCATTCATTTAATTTCTTGTGTTGTATGGATGACTGTCTCTCCTGTCAAAGTTACAGCTGACCCATGGACTTATAAAGATCAAATTATGCTCATCTGTGAAAGGGGGAATACTATAACTTTAACCATAGTTGTGTTCATAGGTTGGTTTCTTGGTTTCCTGTGTCTCCTGTTTTCCTACATGGGAAGAGATCTGCCAAAAAGTTACAATGAGGCCAAATCAATAACCTTTAGTCTCATTTTGTACTATCTGACCTGGATTGTATATTTCACAGCATACCTCTCTTTCAAAAGTAAATACATCACTCTTTTAAATGCAATGGCTCAAATATCCAGTATAAATGGAATTCTCTTTAGCTATTTCATACCAAAATCTTACGTCATAATATTCCAACCACAAAAGAACACTCCCGCATACTTTCAAACATCTATTCAGAAATACACCCAAACCATTAGTAGGATTTAGacattagtctttgtgtatccaccttatttttaacgtaagagcagtgctgccatttgtaacttgaatgtgcaaggcttccggtctcatctgcttccagttattttagctgtataaaaacatgaaattgcaaactggtatttgttatcatattatttgaatcttttatcataattgtaaacacactgatttgtagcgcaaatagttttactgtttactgcacgtggtttcttctaattatttacttatagaagctgatgaatcagaagtctagcacattcacagaaatgagcttactttcgagttgcaaaataaggtggataggatgtgaactcttaaactctgtgttaatggctttgaaggctgacatgctaacatcgctgctgaagaactgtgctacactactaccttcaataaattcttctcccacaagaagtcttgtcaagcttacttattttaggtattagagaaatctaatacattggagagccacccaaatgactgctcggccaaatacagcaaaatctaacaattggcttagatccttctcttttaagttaccacaatcttaaagaaacacataggcaaggtaaagaatggagatctattctgaggttcagtctaaattaagatactatatgtgaattttgggaataaaaagaatttaaaaagtaataaaaagcaatttaaatatttataaaacttttgtttacatgctagtattaacacttaagtgtaatataatgtaaacacaaatgcttgtttttaacttgtaataaagaacaccttcaaaacttccataacttagcgtccaggtaatacatagatttgtgtggtttgaatgtttgtcatcatactgactgcctattcaaactcatacgtgtaacagtatgtaatagtctaatacatttaagtatactattagtaacaatacttttttcattacaaattgttttatcagcattacatttttgaacctgcaatttttttttttagaatgtcc

>gcT1R2F

tgcattcatttgcaatcgtcagagtgtgttgttgtttttgtttgtttgtttgtttcaagagtggaaagatttgggaaaagaagacagtgagcctgcctagggtaaatcaagataccatctaaagagcatggtcggctgcttcaagtggcaacagaatccctaaagccgattggaattcaatcagataaagacatttcatggacatttgaagaaaaaaaatcttttattttattgtatccattacagtcaagctgcgtgtgtctttggtgttgatctaagcttaatttggggtatacacgccaggttatatatacaggattgtgtaataaatgccaagtgttctagtaatccaattgtgcctcaggtttaatactgttccacaaacctagagggatgtctagaactgctacacagttgtcatatgatttctgtgacacttgtggggtgacaaatctacagtaatgacaggcatgcaaaaacatttaaatgtatgcaggaactgggttaagcaaatttaaaaatgtaaagtaaatgtgaacagcaatagtgttgctggccattcaaaacctatgaattcagcactggttttcagcctccatggagagcattaactatattttatactcaaatgtgatgtgtaataccacaatactcaactcaacaaaagctgctgtatacacatcccagagtcattttttatattttctcaccacctctaaacttttaaaccttagtgattgtaatccaaaaaacccctgctgagagtttagaaggttacagtttgtaaaatattttatgaatgtggtgtaatgctctatttaataattgattgacatatgctttgtgagtttagatatggaaatgggcatttgtatcattgaatatggtttaagcaaattgaaatgagttttaagaaaattaagagtagaatataagcctgacactcaaacgactagtcactgcacatccctgttttatgaccaaaaggccatcttaaacaATGTGTCTCAGTAGCATTTACATTTTCCTTGTGGGCTTTACTAATTGTTTCTTTTTCAAAGTTTCTTGCTCAAAAAGTGAGTTCAGCTTGGAAGGAGATTACTTATTGGGTGGCCTTTTCGCTTTACATGAAATTGAACAAGCAACACCCCTGTTCTCCCCAGAGACCACTGAATGTTTCAGgtaagcaaggatttttttatcagctgggttatcaatatgcaatgttgtaaatcaaagcagcatttcctcaaagtcaaatttataaaatgaaaattaaattacatgctattaacttcctcactgatgttttcaagGCACCGTTCCTCAAAATCTGGCTATCAGATGATGCAAGTAATGAGGTTTGCTGTTGAGGAGATTAACAACTCCACCACTCTTCTGCCCAATGTTTCTCTGGGCTATGAAATTTTTGACCATTGTTCTAATACAAAGAATTTCCCTTCAGTCTTAAGTTTTATCTCAAAGAATGGATCAATAAAACCTAAAGAAAAACTCAACAACTATCAGCCTAAAGTGATTGCTTTAACAGGGCCATATGGAAGCACACCAACTATTACTATTGCACCACTGATCACAATGGACCTTATACCATTGgtaaattgtttctttttatacagtttacataacaaaatgcttacacacttactccctaccctttgttcaggactaccaagttctctctctctctctctctctatatatatataatgctaatgaattgtttatagtttagtgttttcttctccttcaaagGTGAATTATGGAGCTTCTACCTCTGCGTTAAGCAACAAACTAAAGTATCCCTCTTTTGTAAGAACAATCCCTTGCAACAAAAACCTGATAGAGATGATTATTCACATCATACGGTGGTTTGGATGGAACTGGGTTGCCTTTCTTGGTAGCCAAGATGATTACAGTTCAGACGCACTAAAGCTGTTTAACGAGTATATAAGTAATACTAGCATTTGTTTGGCTTATCAAGAGAGTATAAGCCTAAACACAAACTACAATCTAACATTTCAAAAGATTGATATGCTCAAGATCAATGTCATTGTTGTTTTGGCTCTGTCACAATATGCAAGCAAAATTATCAAAGCAGCCATAGCAAACAACATCCAAGACAAAGTATGGATTGCAAGTCAGTCATGGGCGATGAATCAACAGATTCCCAGAGAGCCAGGAATTGGGAAAATTGGCACAGTCATTGGTATTACGGAGAGATTGTTGTCAGTGCCTGGATTTAATGAATTTGTCTATAAAGACAGGAGAACAACTGATGTTAACCATGATGAAGAGGGTGACATCCAGAGTACAAGAAAGATGTGTAATCAGGTTTGTGATTACTGCACATTGTTGACCCCAGAGGAGATTATAAATGAAAATCCCACATTATCCTTTGCCATCTATTCTGCCATATATACCATAGCTCATGCTTTACATAAGGTTATGCAGTGTGACATGAATGAATGCCGCAAAAACACAGCGGTTAAGCCATACATGgtgagtgaattcatttgaatccatgacaagttgaatcattgttttaattacattattgaataaccatgttttaattaattaaataatgaataatattgttaatatttttatgaaattgcatattatattatttttatataataatttaataaatgaataaaacaaaggtaatattcatttaactaaagttatagtaatattttactttttttttgttgttgttaaaaaataaataaataaatcaccacaacttacaacagCTTCTGGGAGAAATAAAGAAGTTGGATTTTCCACTCAGTGGCCGTCAGGTGAAATATGATGATAATTATGATCCAACCATCAGTTATGCAGTTGTGCTCTGGCACACTGATGTGAATCCTCCACAGTTTGAGATGGTGGGCACGTATGATACATATCCAGAAGTGACTTTTACCATCAACAACTCTCTCCTGCCCTGGCATAACAATGGTTCTgtaagtcttaatttgataagcctttatctaaacatttaactttatagattccagtttttctcaatcagttttgaaatactgacatcaagactattacaacccgaattccggaaattaaatgttcaaatgtttaaatttgaataaaatgaaaactaaaagactttcaaatcacatgagccaatattttattcacaatagaacatagataacataacaaacgtttaaatagagaatttttacacttttatccactaaattagctcatttcaaatttgatgcctgctacaggtctcaaaaaagttggcacgggggcaacaaatggctgaaaaagcaagaaattttgaaaagattcagctgggagaacatctagcaactaattaagacaattgacatcaggtctgtaacacaattagctataaaagggatgtcttagagaggcagagtctctcagaagtaaagatgggcagagctgtgaaagagtgagtaaaaagattgtggaatattttaaaaacattgttcctcaacctcaaactgcaaaggcttttattaaataaaaaaaaaaaactgggcataataaagaaactgcgtcgtggtaatggaatcccagtttcaaatcaccccggtcgataatcaaacaaatgcacaaaactacttaaggaaaatgattgaatttattaaattcattaaattattaaatttattaaaaataaatataacaagtgaaagggagcatctcttcagggtgtccaaaggccaaaataacaaacaaaacaaatctagaaaggaaaagagggagggattaaataacctaagtaaatataaaaataaaatacatttaccttccctaactccctaacgaaaaacaaagtacaaaccaagttaccaaaataaataggcaggacacccctatgctccacgttcttgaaacataactttaggaacagttgaacatgaccaacaggttgtacaatcggtacactttcacttggaagtaacaagtttgataatcggccggaggagggatccaggatctctcaacccaaacgtggccaaaccaacaatgagcttcatgttaaagaccacgcctcctctgaaccgccaatagagaaagtgtacaccatcactggacaacctactagaacactgacagaacaagagggaaaagaaaacaaacaacacataccaaatacaagaacgcaggttgtaagactttgcaaatctcatcatctacagtgcataacatcatcaaaagattcagagaaactggagaaatctctgtgcataaggaacaagaccgaagacctttattggatgcccgtggtcttcgggccctcagacgacactgcatcactcatcggcatgattgtgtcaatgatattactaaatgggcccaagaatactttcagaaaccactgtcggtaaacacaatccgccttgccatctgcagatgccaactaaagctctatcatgcaaaaagaaagccatatgtgaacatggtccagaagcatggtcctgtttcaaagtggaaacgtgttctatggtcagacgagccaaaatttgacattcttgttggaaatcaccgtgaaatcatttcagggaaggccttgtgtatttcagcaggacaatgcaaaaccacatactgcagctattacaacagcatggcttcgtcgtagtagagtccgggtgctgaattggcctgcctgcagtctagatctttcacctataaagaaaaaaacgtcaaagacgaccacaaactcttcagcagctggaaacctgtatcaggcaagaatgggaccaaatttcaacaccaaaactcctgaaactcataacccagacatcttcacactgttttgaaaagaagaggagatgctacaccatggtaaatatgcccccgccccaactattttgagatatgtggcaggcatcaaatttgaaatgagctcattttgtgcataaaattgtaacatttctcactacatttgtacatttgttatgttatctatgttctattgtgaatcttttagttttcattttattcaaatttaaaaaacgtcccaacatttccggaattcggtgtgtagattgttctgttgactgcatatgcatgtatactactgaaaaagcacatttatcataaagctgacaaaagatctttttttctatgtttgaaagcaagatctgagaaatttgtgggtaggaaattatgtgatttgtattttgtaattcggaatggaaattgaatatttttcagatggctgagttgtttgagaatattgactccagtttagagaaatgtgtaaaatcagttgagaaaaactttaaatttaatgaaacattttcctattcctttaagGTTCCTTTCTCAAACTGCTCTGCTGAGTGTAAGGAGGGATTTTCAAGGCAACCTGAAGGTTTTCATAGTTGCTGTTTTACGTGTAAAAAATGCCCATACAATAGCTATGTGAATTATTCTCgtaagtacaaattaagtatccaaaaacattacatgcagttcaaatttttaatttatttttttttgttgtaattttcacaagcaagtgttacattttcaaaactcaaagtaatctcagaaaaaacattaaaattgcactttatttgtgattttttatttattagacattttaagtttataactatttacaacaaagcccaagatctgcccacaatcataagctataaagataagacttaataaaattaaaataaataataaataaataaacgccgtgcactttagctgtgctgcactccaaaagggcaatgtccagcaagtaggacatccaaatggacatagctggggtagatggttccaaccacaatttcaggatgacctttttatcagcagtaagagcagcggacagagtcctcttttgattgacagacagtgacagcgaagcatcatctagcaaaagaaaaaccaaaaaggaggtcccagaacatatgcatatacaccgatcaggcataacattatgagcactgacaggtgaagtgaataacactgattatctctttatcacggcacctgttagtgggtgggatatattaggcagcaagtgaaattttgtcctcaaagttgatgtgttagaagcaggaaaaatgggcaagcgtaaggatttgagcaagtttgacaagggccaaattgtgatggctagacgactgggtcagagcatctccaaaactgcagctcttgtggggtgttcccggtctgcagtggtcagtatctatcaaaagtggtccaaggaaggaacagtggtgaaccggtgacagggtcatgggcggccaaggctcattgatgcacgtggagagcgaaggctggcccgtgtggtccgatccaacagacgagctactgtagctcaaattgctcaagaagttaatgctggttctgatagaaaggtgtcagaatacacagtgcatcacagtttgctgcgtatggggctgcatagccgcagaccagtcagggtgcccatgctgacccctttccaccgccaaaagcaccaacagtgggcacgagagcatcagaactggaccacggagcaatggaagaaggtggcctggtctgatgaatcacgttttcttttacatcatgtggatggccgggtgtgtgtgcgtagcttacttggggaatacatggcaccaggatgcactatgggaagaaggcaagccgacggaggcagtgtgatgctttgggcagtgttctgctgggaaaccttggatcctgccatccatgtggatgttactttgacacgtaccccctacctaagcagtgttgcagaccatgtacaccctttcatggaaatggtattcactggtggctgtggcctctttcaggaggataatgcaccctgccacaaagcaaaaatggttcaggaatggtttgaggagcacaacaacaagtttgaggagttgacttagcctccaaattcctcagatctcaatccaatcgagcatctgtgggatgtgctgaacaaacaagtcctcacaacttacaggacttaaaggatctgctgctaacatcttggtgccagatatcacagcacaccttcagggtctagtggagtccatgcctcgacaggtcagggctgtattaggtgtggtcataatgttatgcctgattggagtatgtacctggacttgcactgctacacaaaattgcactttttttttttaaagtttcagcatattttaaatgatgttaatagaaaacacaaaatcaaagtatccttttcactacagaaaataagtgtttaatctatataaatgtttaattcacagtaactgcctttcataatgctgttactactataatgctatcttttcattagcatctcttcttgtttagtttaatacatttgtctatcatttaatccaactgatcttaatatttaatcgatgaatcatttggtacatctatacattctataaaagtggcttgcaatttccaaaacggataactaaatataaggaaataattacagaataagcaatttatctcacatgaaaaccactggtgtcaaaagtattcacattcattaggtagaagtatagatactagggtttaaaaaagatttctgtagaagttgaaatatcaactcaagctttttactcaagtaaaagtgtaaaagtactggtttcaaaactacttaaagtataaaagtaaaagtaatgtaagggaaaaaaatgtcattaaggacaaaagcttaggccgcgccacaggggcctattgtgcactaccccacccccaccacctcctcaaaaaaacatttttctaaaggccatataatgactataatgttatattaaaatgttaatgttgaaaaatttgggatacactaggctacctgtttgtaccaacctcctcgctggtgcttggttgggacattttgctcgagttctcttcttgagtctctgccacggacatcttcgtacttcgctgcataagtctgctatgtccagtgttgggggtaacgagttacaaagttagtatagcctacttatcacaacattgtcaatcgcaaacgtcaattcaaacgtctctctaccgaactgcctactgtagcttaatttgcggaggacgaagagaaagcacccgtttgataaataagccagtagtggagaaataataacttttaagaaataatcttttttgagtaacgaccccaacactggctatgtcctccttgctggagtgtgacgtgatttgtgtcatgtgcaggtgcgatggatcgcgtacaaaccaatagggtgtcagaatggtatatgtttatacttctcatccaaccacaatcaaattcactttatccggatggcgcgatttatctggataggttttttttttttttttgtggggatatttttggttcccatgaggaaaacaactttaaatcatactgtggtgacttaaattatgtttttttgaaaatctaaaatatcagaaatttttctgtggtgggtaggtttaggggtggggttagtgtaacctcatggccatatcatatcttcttcaaatcttttaatctcacactggatacataccatctcaaacattatgtattttactttaaaggcacaatgtgtaagattttttggattaaaatatcaatgttatattttgttgacttatgtacttatcccaaatgtttccaagaatgtttaaatccagagaaataagcaattttaaccaggacacggactgtgtccgtgcgtcgcctatcaatgacccgcgttaccctcgatttcctcaactatttggatggatacattcattgacagaaaactaatcatgttatatagctcaacacaggaagtcttattgtttagatcttgtttgctagcttactgcagtgtgcattaagtgtctcatagcagccgccgagcaaacacagcgaaacaactttccgcacacaaatgtatctactatgataaaaaagttccgctgctctcgagccgtgtgtcgcgctcgtctctcattagcaatcactccagcggtctcattcagctcccacagcactcggccctgctctgcttcatactacagtaacattaataatctcatccacgaacatgacttctgcccgagtcccatcccgattcttttccactggctgtagacatgaagacaacccctcccatgattccgcggcatcaagctacatctttgttttgaataagcgacctctagtggtgaaaatttacatattgtgcctttaagtcagttacatgttaggtaatgtgtgtattttttgaatgcgacaactgtagcctgtattactgtaatgtctttaattcagcaacagggctgatttgaaatatgtatcttgcattgcttactattgaatgaactgattgctaaaactactacactgaaagaagatcattatgttctggtgatttaaccaaatgttctcattatatattagaatgagcttgtgttttgaaacaattattgtgtagaaaaaaaaaatggtatatttgctgtgctagactaactgagacttgttacagcacttgtatacagttgccctcttattggtctgattgcttctattgttcttctcatttgtaagtcgctttggataaaaccgtctgctaaatgactaaatggaaagaaaaaaatggaaagaaaacatgtatagattttgaaagaatgactagctgtgttacaagtgtgaacaatttggatttttgtaggttagaatttccaccttatgaaaatagtacaaaagtgaagtatattcttttagtgtactgtacttctttttcacaaagcttactattataaactttcatattattttttcagGGGACCCCTATACCTGTTTTCCATGTGCAGAGAATGAATGGTCTGATGAAGGCAGCACAACATGTAAGAGACGTTCTGTTGTCTATCTTCAGTTCACAGAAATCCCCTCCATCGCTGTCTTGGTTTCTGCCATATGTATAATTATTTTACTCATTGCCATATTTTGTATTTTCGCCTACAATTACAACACACCAGTGGTGAGGTCAGCTGGTGGTAGCATGTGTTTCCTGATGTTGACGTGTTTGATTTTGTCTAGTATAAGTGCATTCTTTTTCTTTGGAGAACCCACATCTGCACTTTGCCTCCTGAGAAATGCCATATTTGCATTTTTCTTCACTGTCTGTATTTCCTGTTTGACTGTCCGTTCCTTTCAAATTGTTTGTGTTTTTAAAATGGCTGCTCAGTTCCCTAAGGTGCACAGCCTTTGGGTAAAGCACAATGGCCAGTGGCTCTTCATTGCATTGGTGTCTGTCATTCATTTAATTTCTTGTGTTGTATGGATGACTTTTTCTCCTGACAAACCCATTGCCGACTCATGGACTTTTAAAGATCAAATTATGCTCATCTGTGAAATGGGGAATGCATTAACCATAACCATAGTTGTGTTCATAGGATGGTTTCTTGGTTTCCTGTGTCTCTTGTTTTCTTACATGGGAAGAGATCTGCCGAAAAGTTACAATGAGGCCAAATCAATAACCTTCAGTCTAATTTTGTACTATCTGACCTGGATTGTATATTACACAGCATACCTCTCTTTCAAAAGTAAATACATCACTCTTTTAAATGCAATTGCCCAGATATCTAGTATAAATGGAATTCTCTTTAGCTATTTCATACCAAAATCTTACGTCATAATATTCCAACCACAAAAGAACACTCCCGCATACTTTCAGACATCTATTCAGAATTACACCCAAACCATTAGTAGGACTTAGacattagtctttgtgtatccaccttatttttaatgtgagagcagtgctgccatttgtaacttgaatgtgcaaggcttccagtctcatccacgtccagttattttagctgtacaaaaacagtcctttatgctgacggatattgctagtatttatcatattatctgaatctgttatcgtaatcataaacatgatttgtatcacaaatagttttaccgtctactgcactttgttattcttctagttatttagaggctaatgaatgggaagtctagcacattcacagaaagtagcttacttttgagttgcaaaataaggtggatatgtgttaactcttaaactctgtgttaatggcttagattcttcacttttatgttaccacaatgttaaagaaacgcataggcaaagtagagaatctgttctggggttcaatctgcattaagataatatgtgatttttgagaataaaatgagtaattgacatataaaaagcttttgtttacatgttcgcgtcaacactaatgtaacaatgtaatgtaaacaatttttgtttctcttctttttttaaacttataataaagaacactttcagaacgtccataatagtgtccaattgttacattacttgttttcaaaagtaatattattactttacttattacttctgccaagagtgtttttttacactacttgttatattacttttccgttacaccccataaaatttgtaattgtgttcttttttcctcaaaaattctaacttcacatgtgcatctcatacatatataaatgatgtgaacacatccacctggaagtttggagtatggctagaaagaccttgattagatggttcaggtgtgtttaattggggttggagctaaactctgcaggacagtggccctctaagagcaggattggacaaccctggtttagatggactactaaacatgaaaaagagtggactgacctgaaagcaacatttaccacagagtctgtactgacct
