# Supplementary material for: Expansion of sweet taste receptor genes in grass carp (Ctenopharyngodon idellus) coincided with vegetarian adaptation
Source: BMC Evol Biol. 2020 Feb 11;20:25. doi: 10.1186/s12862-020-1590-1 (PMC7014666; doi:10.1186/s12862-020-1590-1)
Supplement: Supplementary file 2 — Additional file 2: Table S1. Primer sequences. The primer sets for T1R2s sequencing and functional studies. [file 12862_2020_1590_MOESM2_ESM.docx]

**Table S1.** Primer sequences

| **Gene name** | **Primer** | **Sequence 5′-3′** | **AL(bp)^a^** | **AE(%)^b^** | **Tm(°C)** |
| --- | --- | --- | --- | --- | --- |
| **Primers of T1R2s sequence verification** | | | | | |
| gcT1R2A | T1R2A-F | ATGTTTCACAGTAGCATTTACGTTTTC | 2496 |  | 58 |
|  | T1R2A-R | CTAAGTCCTACTAATGGTCTGGGTG |  |  |  |
| gcT1R2B | T1R2B-F | ATGCTTCTGAGTAGCATTTACTCTTTCC | 2502 |  | 58 |
|  | T1R2B-R | CTAAGTCCTACTAATGGTCTGGGTG |  |  |  |
| gcT1R2C | T1R2C-F | ATGTGTCTCAGTAGCATTTACATTTTCC | 2496 |  | 58 |
|  | T1R2C-R | CTAAGTCCTACTGATAGTTTGGGTG |  |  |  |
| gcT1R2D | T1R2D-F | ATGTGTCTCAGTAGCATTTACATTTTCC | 2490 |  | 58 |
|  | T1R2D-R | CTAAGTCCTACTGATAGTTTGGGTG |  |  |  |
| gcT1R2E | T1R2E-F | ATGCTTCTCAGCATTTACATTTTCC | 2493 |  | 58 |
|  | T1R2E-R | CTAAATCCTACTAATGGTTTGGGTG |  |  |  |
| gcT1R2F | T1R2F-F | ATGTGTCTCAGTAGCATTTACATTTTCC | 2496 |  | 58 |
|  | T1R2F-R | CTAAGTCCTACTAATGGTTTGGGTG |  |  |  |
| \| **Primers of recombinant T1Rs plasmid construction** \| \| \| \| \| \| \| --- \| --- \| --- \| --- \| --- \| --- \| \| gcT1R3 \| gcT1R3-F \| ATGGCTAAGGAGTGGACGCTT \| 2538 \|  \| 58 \| \|  \| gcT1R3-R \| CTAGCTTTCTTCAGGTGGTGTTGG \|  \|  \|  \| \| zfT1R2a \| zfT1R2a-F \| ATGCTTCTTGATAGCAATTACATTTTCC \| 2478 \|  \| 58 \| \|  \| zfT1R2a-R \| CTAAGACCTACTAATGGTTTGAGTG \|  \|  \|  \| \| zfT1R2b \| zfT1R2b-F \| ATGCTTCCATGTTACTCATTCCTTTTG \| 2472 \|  \| 58 \| \|  \| zfT1R2b-R \| TTAAGTTCTACTAATGGTTTGGGTG \|  \|  \|  \| \| zfT1R3 \| zfT1R3-F \| ATGCTTCTACTGAGGATGAAGAAC \| 2553 \|  \| 58 \| \|  \| zfT1R3-R \| TTAGCTTTCTTCAGGTGGTGTAGGTG \|  \|  \|  \|   **Primers of T1R2s sequences for the quantitative real-time PCR** | | | | | |
| gcT1R2A | rtT1R2A-F | GCATTAGATGGAGGATGATTG | 273 | 101.1 | 56 |
|  | rtT1R2A-R | GATTGTGGTAACATAGTAGTGAAG |  |  |  |
| gcT1R2B | rtT1R2B-F | CCCAGACCATTAGTAGGACTTAGA | 210 | 98.1 | 59 |
|  | rtT1R2B-R | AACAAAACTCAAATACAACACCAC |  |  |  |
| gcT1R2C | rtT1R2C-F | CCAACCACAAAAGAACACTCCT | 235 | 99.1 | 56 |
|  | rtT1R2C-R | ACTTCAGACATCGTCACGCCAA |  |  |  |
| gcT1R2D | rtT1R2D-F | TCCAACCACAAAAAAACACTCC | 133 | 100.3 | 56 |
|  | rtT1R2D-R | TTCAAGTTACAAATGGCAGCAC |  |  |  |
| gcT1R2E | rtT1R2E-F | CACCCAAACCATTAGTAGGATT | 126 | 102.5 | 57.5 |
|  | rtT1R2E-R | ATAACTGGAAGCAGATGAGACC |  |  |  |
| gcT1R2F | rtT1R2F-F | GTGCTGCCATTTGTAACTTG | 226 | 97.8 | 57.5 |
|  | rtT1R2F-R | GACTTCCCATTCATTAGCCTCT |  |  |  |
| **Primers of candidate reference genes sequences for the quantitative real-time PCR** | | | | | |
| β-actin | rtβ-actin-F | AGAGGGAAATCGTGCGTGAC | 193 | 102.0 | 60 |
|  | rtβ-actin-R | ATACCGAGGAAGGAAGGCTG |  |  |  |
| RPL13A | rtRPL13A-F | CTGAAGGAACAGCTCAAACAA | 155 | 101.5 | 58 |
|  | rtRPL13A-R | GGAGAAGAGGAAGGAAAAGG |  |  |  |
| EF1 | rtEF-R | GCTGACTGTGCCGTGCTGAT | 201 | 98.6 | 58 |
|  | rtEF-F | GCTGACTTCCTTGGTGATTTCC |  |  |  |
| TUA | rtTUA-F | TACGAACAAGAGCGAGATG | 177 | 99.3 | 58 |
|  | rtTUA-R | AGTGATGATGGAGAGAGAAAG |  |  |  |
| GAPDH | rtGAPDH-F | ATGCCTCCTGCACCACCAA | 149 | 100.2 | 58 |
|  | rtGAPDH-R | ATCCCTCCACAGTTTCCCA |  |  |  |

^a^AL, amplified length of PCR production (bp);

^b^AE, amplified efficiency of primer (%).
